# CD4 Inhibits Helper T Cell Activation at Lower Affinity Threshold for Full-Length T Cell Receptors Than Single Chain Signaling Constructs

**DOI:** 10.3389/fimmu.2020.561889

**Published:** 2021-01-19

**Authors:** Deborah K. Johnson, Wyatt Magoffin, Sheldon J. Myers, Jordan G. Finnell, John C. Hancock, Taylor S. Orton, Stephen P. Persaud, Kenneth A. Christensen, K. Scott Weber

**Affiliations:** ^1^Department of Microbiology and Molecular Biology, Brigham Young University, Provo, UT, United States; ^2^Department of Chemistry and Biochemistry, Brigham Young University, Provo, UT, United States; ^3^Division of Laboratory and Genomic Medicine, Department of Pathology and Immunology, Washington University in St. Louis, St. Louis, MO, United States

**Keywords:** CD4, T cell receptor, affinity, chimeric antigen receptor, Lck, helper T cell

## Abstract

CD4^+^ T cells are crucial for effective repression and elimination of cancer cells. Despite a paucity of CD4^+^ T cell receptor (TCR) clinical studies, CD4^+^ T cells are primed to become important therapeutics as they help circumvent tumor antigen escape and guide multifactorial immune responses. However, because CD8^+^ T cells directly kill tumor cells, most research has focused on the attributes of CD8^+^ TCRs. Less is known about how TCR affinity and CD4 expression affect CD4^+^ T cell activation in full length TCR (flTCR) and TCR single chain signaling (TCR-SCS) formats. Here, we generated an affinity panel of TCRs from CD4^+^ T cells and expressed them in flTCR and three TCR-SCS formats modeled after chimeric antigen receptors (CARs) to understand the contributions of TCR-pMHCII affinity, TCR format, and coreceptor CD4 interactions on CD4^+^ T cell activation. Strikingly, the coreceptor CD4 inhibited intermediate and high affinity TCR-construct activation by Lck-dependent and -independent mechanisms. These inhibition mechanisms had unique affinity thresholds dependent on the TCR format. Intracellular construct formats affected the tetramer staining for each TCR as well as IL-2 production. IL-2 production was promoted by increased TCR-pMHCII affinity and the flTCR format. Thus, CD4^+^ T cell therapy development should consider TCR affinity, CD4 expression, and construct format.

## Introduction

CD4^+^ T cells are critical for tumor elimination through both indirect and direct mechanisms. Indirectly, CD4^+^ T cells target tumor cells by activating tumor-killing cells such as CD8^+^ T cells, macrophages, B cells, and natural killer cells (1–4). CD4^+^ T cells have direct cytotoxic effects against tumor cells that express major histocompatibility complex II (MHCII) (1–4) and direct CD4^+^ T cell responses are less toxic to the patient than a CD8^+^ T cell response, especially when responding to overexpressed tumor associated antigens (TAA) (5). The presence of tumor-specific CD4^+^ T cells is correlated with improved patient survival following vaccination with cancer-associated peptides whether or not they are directly involved in tumor suppression (6–8). Furthermore, CD4^+^ T cells can sustain an immune response when CD8^+^-specific antigens are lost which otherwise might result in tumor escape (9). Despite these clear benefits, only one published clinical study (10) focuses on the immunotherapeutic benefits of CD4^+^ T cell receptors (TCRs) (10, 11).

CD4^+^ T cells are activated by interactions between the TCR and its cognate peptide presented on MHCII (pMHCII) (12). TCRs can detect a single amino acid change and distinguish between self-proteins and mutated neoantigens (11), uniquely suiting TCR-based therapies for specific tumor targeting. Furthermore, unlike antibody-based chimeric antigen receptors (CARs), which are limited to extracellular targets, TCRs can target intracellular antigens presented by MHC molecules (11). To rationally design optimal targeting strategies, it is essential to understand how the TCR:pMHC interaction impacts T cell responses. The relationship between TCR affinity and T cell activation is complex, but in general, T cell functional activity correlates with TCR binding affinity for pMHC (13–19). However, there are important nuances to this general theme. For example, tumor-associated antigens may be skewed towards lower-affinity clones due to thymic negative selection (20, 21), even the lowest-affinity TCRs can induce T cell proliferation, cytokine production and memory formation (19, 22). On the other end of the spectrum, high affinity TCRs have been shown to enhance immune responses in some cases (23) and attenuate responses in others (24–29), with some reports showing evidence of an affinity threshold beyond which increased affinity does not impact the magnitude of the response (17, 18). An additional consideration is that even when high-affinity TCRs are capable of heightened cytotoxicity and tumor control, these TCRs may be predisposed to autoimmunity (30). Thus, the optimal affinities for TCRs engineered against tumor-specific peptides may lie within a low or intermediate affinity (14, 24–26, 30–36). As most affinity studies to date have focused on CD8^+^ TCRs, CD4^+^ T cell affinity thresholds are less well characterized.

The role of the CD4 coreceptor is an important consideration when associating TCR-pMHCII affinity to CD4^+^ T cell activation. CD4 binds to MHCII as part of the TCR complex and contributes to proximal TCR signaling, proving especially critical for T cell function when cognate pMHC ligands are limiting (<30 complexes) (37). TCR signaling dependence on CD4 is affected by the quality of TCR:pMHCII interaction and is unnecessary upon stimulation with optimal ligands (38). Thus, CD4 may be restricted to improving the TCR dwell time on pMHCII for lower affinity interactions (39). As CD4^+^ TCRs can function in natural killer cell lines without CD4 (40), CD4 may not have as great of an effect on T cell activation as CD8, particularly with high affinity TCRs.

To determine how TCR-pMHCII affinity and CD4 coreceptor interactions affect CD4^+^ T cell activation, we examined activation of the CD4 transgenic murine T cells LLO118 and LLO56 that are stimulated by the same *Listeria monocytogenes* epitope. These TCRs differ by 15 amino acids and recognize the LLO_190-205_ peptide presented by the MHCII molecule I-A^b^ with similar affinity (41, 42). LLO118 has a more robust primary response and LLO56 has a more robust secondary response, indicating that TCR affinity is not the only parameter affecting activation in these cells. To examine the role of affinity in the activation responses of LLO56 and LLO118, we engineered an affinity panel of CD4^+^ TCRs (ranging from 4 µM to 200 nM) using yeast display (43, 44). After characterizing their affinity and avidity, the activation characteristics of two low affinity clones, two intermediate affinity clones, and one high affinity clone were examined in the full length TCR (flTCR) format or in three TCR-SCS CAR formats (CD28- and 4-1BB-based second generation CARs, and CD28/4-1BB third generation CAR). T cell receptor single-chain signaling chimeric antigen receptors (TCR-SCS CARs) are an exciting potential therapeutic option and as CD4^+^ T cells are potent responders to cancer, we sought to understand how CD4^+^ TCRs respond to a variety of affinities. TCR-SCSs constructs avoid mispairing with endogenous TCR chains, which is an inherent risk for engineered flTCRs (45). CARs also produce more cytokines and are activated by higher antigen densities than flTCRs and may be more likely to ignore healthy cells with low amounts of TAAs, which may improve clinical outcomes (46–48).

We found that increased TCR affinity promotes production of IL-2 regardless of flTCR or TCR-SCS format. The flTCRs are more responsive to lower amounts of peptide stimulation, and contrary to CD8^+^ TCR findings (49), produce more cytokine than TCR-SCSs. While there are some observable trends dependent on second and third generation TCR-SCS CAR format, IL-2 production varies depending on whether the TCRs were engineered from the LLO56 or LLO118 TCRs. CD4 promotes the activation of low affinity flTCRs and TCR-SCSs, but CD4 is inhibitory in intermediate affinity flTCR and high affinity TCR-SCS CARs. The flTCR reaches CD4 inhibition at a lower affinity than TCR-SCSs, suggesting that flTCRs perceive a stronger initial activation signal. These findings suggest that therapeutic CD4 TCR development should consider construct features, TCR affinity, and coreceptor activation contributions when choosing or engineering therapeutic TCRs and cell lines.

## Materials and Methods

### Library Construction

Yeast display allows for the external presentation and screening of large libraries of genetically altered proteins (43, 44). Single chain T cell receptor (scTCR) contain linked variable TCR α and β domains but lack constant domains and any signaling domains. The scTCR constructs for LLO56 (residues 1–116) and LLO118 (residues 1–120) (Invitrogen) consist of the mature Vβ domain, a 13-aa linker (DAKKDAAKKDDAS) (50), followed by the mature Vα domain (LLO118 residues 1–112 or LLO56 residues 1–113), and an N-terminal HA tag (PYDVPDYA). To display scTCRs on yeast, the constructs were placed in pCT302 (NheI and BglII) (Addgene plasmid # 41845; http://n2t.net/addgene:41845; RRID : Addgene_41845) (51). Stability clones were selected from scTCR transcripts replicated by error-prone PCR (Standard Taq, New England BioLabs, B9014S) (44). Affinity libraries were generated using site directed mutagenesis of 5 amino acids in the CDR3β region and splicing by overlap extension (SOE) PCR (44, 52) using LLO118 and LLO56 specific primers (Q5 High Fidelity DNA Polymerase, New England BioLabs M0491) (**Supplementary Materials**).

To generate yeast libraries, 150 ml cultures of growth phase EBY100 yeast were collected and washed twice with 50 ml ice-cold water and once with ice-cold electroporation buffer (1 M Sorbitol/1 mM CaCl2) then resuspended in 0.1 M LiAc/10 mM DTT and incubated at 30°C and 225 rpm for 30 min (53). Cells were washed with 50 ml electroporation buffer, resuspended in 200 µl electroporation buffer and aliquoted with digested pCT302 backbone (NheI and BglII, 1,250 ng) and inserted (6,250 ng) into 0.2 mm gap cuvettes then electroporated (2.5 kV and 25 µF). Cells were allowed to recover for 1 h in 4 ml 1 M sorbitol:YPD media (1:1) and were resuspended in SD-CAA media and incubated for 2–3 days at 30°C before quantification. Stability and affinity library sizes ranged from 1.1x10^7^ to 1.9x10^9^.

### Stability Clone Selection

Libraries calculated to have at least 10 copies of each clone were placed in 5 ml SG-CAA media for 36–48 h to induce scTCR expression (54). To select stability clones, yeast libraries were incubated with either 2 µg/ml anti-mouse TCR Vα2 or anti-mouse TCR Vβ2 phycoerythrin-conjugated antibodies (BioLegend, clone B20.1 and B20.6, respectively) in 5 ml PBS 1% BSA for 2 h at 4°C, washed with 15 ml PBS 1% BSA and stained with 50 µl anti-PE MicroBeads in 2 ml PBS 1% BSA (Millitenyi 130-048-801) for 20 min at 4°C. Labeled clones expressing properly folded Vα or Vβ were positively selected in magnetic LS columns (Millitenyi 130-042-401). Selected cells were grown in 3 ml SD-CAA media (48 h) before induction in SG-CAA (36–48 h). Each library was subjected to three rounds of growth and sorting, and the most stable clone identified *via* flow cytometry (BD Accuri C6). Stability clones were used as templates for subsequent stability or affinity libraries.

### Affinity Clone Selection

To select affinity clones, induced yeast libraries were incubated with tetramer (LLO_190-201_/I-A^b^) (I-A(b)CC (NEKYAQAYPNVS), NIH 22201), and sorted like stability clones. To isolate high affinity clones, libraries were exposed to an increasingly strict temperature and incubation regimen. Initially, libraries were subjected to high concentrations of tetramer (13.0 µg/ml), high temperatures (37°C), and long incubation times (3 h), and in later rounds, combinations of lower tetramer concentrations (3.25 µg/ml), lower temperatures (RT or 4°C), and shorter incubation times (1 h) were used to isolate the clones with highest affinity. Each library was column sorted three times. Isolated clones with increased tetramer binding were identified *via* flow cytometry (BD Accuri C6).

### Tetramer Dissociation

Each affinity and stability clone K_D_ was determined through tetramer dissociation (55). Aliquots of 1x10^6^ induced cells were stained with 100 µl of various concentrations of LLO_190-201_/I-A^b^ tetramer (0.152 nM to 12.16 nM) for 1.5 h at room temperature and quantified *via* flow cytometry. Tetramer binding was assessed as MFI of positive population and normalized to the highest recorded MFI using FlowJo. K_D_ was defined as 50% maximum binding concentration (55).

### Tetramer Decay

Half-life (t_1/2_) was determined by staining 3x10^6^ cells of each affinity clone with 6.5 µg/ml of tetramer for 1.5 h at room temperature (56). Samples were washed three times in PBS 1% BSA to remove excess tetramer. Following an initial timepoint measurement, 90 µl of 0.1 µg/ml or 1 µg/ml anti-mouse MHC class II (I-A/I-E) (clone: M5/114.15.2, eBioscience) was added and the decrease of tetramer binding was quantified at various time points (2, 5, 10, 15, 20, 30, 45, and 60 min) by placing 10 µl of cells into 90 µl of buffer and running immediately on the flow cytometer.

### scTCR Expression, Refolding, and Purification

The following protocol was modified from Garcia *et al*[61]. Briefly, scTCR constructs were cloned into pET28a (Novagen) using NcoI and SacI restriction sites. Constructs were expressed in BL21 T7 Express *E. coli* (New England Biolabs) and protein expression was induced for 4 h (0.4 mM isopropyl β-D-thiogalactoside). Cells were lysed with 1 mg/ml lysozyme (ThermoFisher Scientific), 5 mM MgCl2, 1 µl/ml DNase I (Promega), 1% Triton-X 100, and 10 mM dithiothreitol followed by two rounds of sonification (Branson Digital Sonifer) for 1 min at 0.5 s alternations at 40% power. Fifty to 200 mg of inclusion body slurry was dissolved in 1 ml of 7M GnHCl and 10 mM beta-mercaptoethanol. Four hundred ml of 2 M GnHCl, 50 mM Tris-HCl, 2mM GSH, 0.2 mM GSSG, and 0.1% NaAz were dripped into dissolved inclusion bodies for 2–4 h at 4°C. Then 2–2.5 L of 200 mM NaCl, 50 mM Tris-HCl, and 0.1% NaAz were dripped for 24 h (1.5 ml/min speed) at 4°C. Following an additional 24 h spinning at 4°C, the refolded TCR solution was vacuum filtered with 0.22 µm PES membranes (Olympus Plastics), and then concentrated in an Amicon 8400 unit (Ultracel 10 kdal Ultrafiltration Discs) under 55psi N_2_. Once the volume was reduced to 50–100 ml of refolded scTCRs, the samples were again filtered with 0.45 µm CA-membrane and GF prefilter syringe filter and purified by FPLC (AKTAstart) on a HisTrap column (GE Life Sciences). Purified scTCRs were concentrated using Amicon centrifugal filters (Ultra 4 10k) and quantified by Pierce BCA Protein Assay kit (Thermo Scientific).

### Bio-Layer Interferometry (BLI)

BLI experiments were performed with an Octet RED96. Streptavidin (SA) biosensors (FortéBio) were hydrated and equilibrated in 1x HEPES buffered saline (HBS, 50 mM HEPES, 150 mM NalCl, pH 7.2), 2mM CaCl_2_, 1 mM MgCl_2_, 1 mg/ml milk, 0.1% Tween, and 0.02% NaN_3_. SA sensors were loaded with 2.0 µg/ml biotinylated LLO_190-201_/I-A^b^ monomer or DQB_187-101_/I-A^b^ monomer to 1.0–2.0 nm. Loaded biosensors were equilibrated in assay buffer until baseline was achieved. scTCR association was probed in wells with assay buffer (stability clones 2, 1, 0.5, 0.25, 0.125, 0.061 µM; affinity clones 800, 400, 200, 100, 50, and 25 nM, or 20, 10, 5, 2.5, 1.25, and 0.625 nM) with a blank reference-subtraction well for 400–600 s. Ideal concentration range spanned one log above and below the K_D_ where possible; however, this range had to be optimized depending on the sensitivity of the assay, and on the amount of protein available. Matching of sample and baseline imidazole and milk concentrations (through serial dilution of sample buffer into baseline wells) was critical for detection of scTCR binding. Blocking with bovine serum albumin increased non-specific binding while milk efficiently blocked NSB. Dissociation was observed in baseline assay buffer (600–1,200 s). Assays were run at 30°C with a plate shake speed of 1,000 rpm.

Data was collected at 5 Hz, using 20-point signal averaging and analyzed using custom kinetic analysis. Due to non-specific binding at the later stages of the association and dissociation steps, K_D_ was calculated by extracting and selecting the data points from the initial association to determine k_obs_ (2–100 s depending on the affinity of the constructs), plotting concentration vs rate, and then plotting those slopes against scTCR concentration and estimating k_assoc_ from the slope. k_dissoc_ is the slope of concentration vs rate of the dissociation step data (2–100 s depending on the affinity of the constructs). K_D_ was determined by dividing k_diss_/k_assoc_ and t_1/2_ = ln2/k_D_.

### Cell Culturing

All 58^-/-^ T cell hybridoma cell lines were cultured in RPMI 1640, 10% FBS, 2g/L NaHCO_3_ (23.8 mM), HEPES (4.2mM), L-glutamine (3.24 mM), 1% Penn-strep and split 1:5 or 1:10 every 2–3 days. Platinum Ecotrophic cells (Plat E) were cultured in DMEM, 10% FBS, 1% pen-strep, 1 µg/ml puromycin, and 10 µg/ml blasticidin and split 1:4 every other day.

### Retroviral Transduction of T Cell Hybridomas

Affinity mutations were cloned into four possible constructs: full length TCRs (flTCRs), and three TCR-single chain signaling formats based on chimeric antigen receptor (CAR) formats (second generation 4-1BB and CD28 CARs, and third generation 4-1BB/CD28 CAR). Inserts were cloned into pMSCV-IRES-GFP II (pMIGII) (Addgene plasmid # 52107; http://n2t.net/addgene:52107; RRID : Addgene_52107) using MfeI and XhoI (GenScript) (57). All constructs were led by a Kozak sequence and either Vα2 signal peptide (MDKILTASFLLLGLHLAGVSGQ) and an additional Vβ2 signal peptide (MWQFCILCLCVLMASVATD) for flTCRs or high affinity M33 3^rd^ gen CAR signal peptide (MLLALLPVLGIHFVLRDAQA) for all TCR-SCS CAR constructs (58). flTCR constructs have a P2A cleavage domain (GSGATNFSLLKQAGDVEENPG) (59) between Cα2 and Vβ2 domains. All TCR-SCS and flTCR were linked to GFP by an IRES domain and GFP expression mirrors TCR construct expression in cell lines.

Vectors were transfected into Plat E packaging cells grown overnight in 6-well plates with TransIT-VirusGEN (Mirus, MIR 6703). Forty-eight hours later, 1 ml of viral supernatant was mixed with 1 ml of 1x10^6^ 58^-/-^ CD4^-^ or 58^-/-^ CD4^+^ cells in a 6-well plate and spinfected for 2 h at 30°C at 1,000 G (acceleration 6, brake 2). After 48 h recovery, clones Vβ2, Vα2, and GFP^+^ expression was checked by flow cytometry. Clones with under 85–90% GFP expression were sorted 1–3 times with magnetic LS columns (Miltenyi Biotec, 130-042-401) using 10 µl Vβ2-PE antibodies and 10 µl anti-PE MicroBeads (Miltenyi Biotec, 130-048-801) per manufacturer specifications. Clones were checked for TCR expression after each sort round. CD4T^+^ and CD4T^+^ Δbind (60) were cloned into pMIGII (MfeI/XhoI) and retrovirally transfected into existing 58^-/-^ CD4^-^ flTCR and TCR-SCS clones and sorted for >95% CD4 expression (CD4 PE-Cy7, GK1.5, Biolegend) by flow sorting (BD FACSAria II). Twenty-five thousand cells were stained with respective antibodies or tetramer for all affinity and stability measurements and measured with flow cytometry (BD Accuri).

### T Cell Hybridoma Peptide-Specific Activation and IL-2 Measurement

2.8x10^4^ T cell hybridoma clones were incubated with 2.8x10^5^ splenocytes (1:10) isolated from BL6.C57 mice with varying amounts (10^-8^ M to 10^-3^ M) of peptide (LLO_190-205_, GenScript) in 75 µl 58^-/-^ media in 96 well plate for 24 h. IL-2 production was measured using an IL-2 ELISA kit (KIT) and measured on a microplate reader. This study was approved and carried out in accordance with principles of the Basel Declaration and recommendations of Brigham Young University’s Institutional Animal Care and Use Committee (IACUC protocol #18-0708).

### Statistical Analysis

Statistical analysis was performed *via* one-way ANOVA with Tukey’s multiple comparison test (p < 0.05 was significant, no alpha adjustments required). Half-life (t_1/2_) was determined by linear regression between time point 0 and the time point where no tetramer binding was detected (56). To determine the K_D_, we fit the data with a non-linear curve, based on one site-specific binding kinetics (55). EC_50_ was determined with Sigmoidal, 4PL, X is log(concentration) least squares fit. Standard deviation is reported for each value. All analyses were conducted in GraphPad Prism.

## Results

### Yeast Displayed TCR Panel Has Varied Affinities

**Figure 1:**
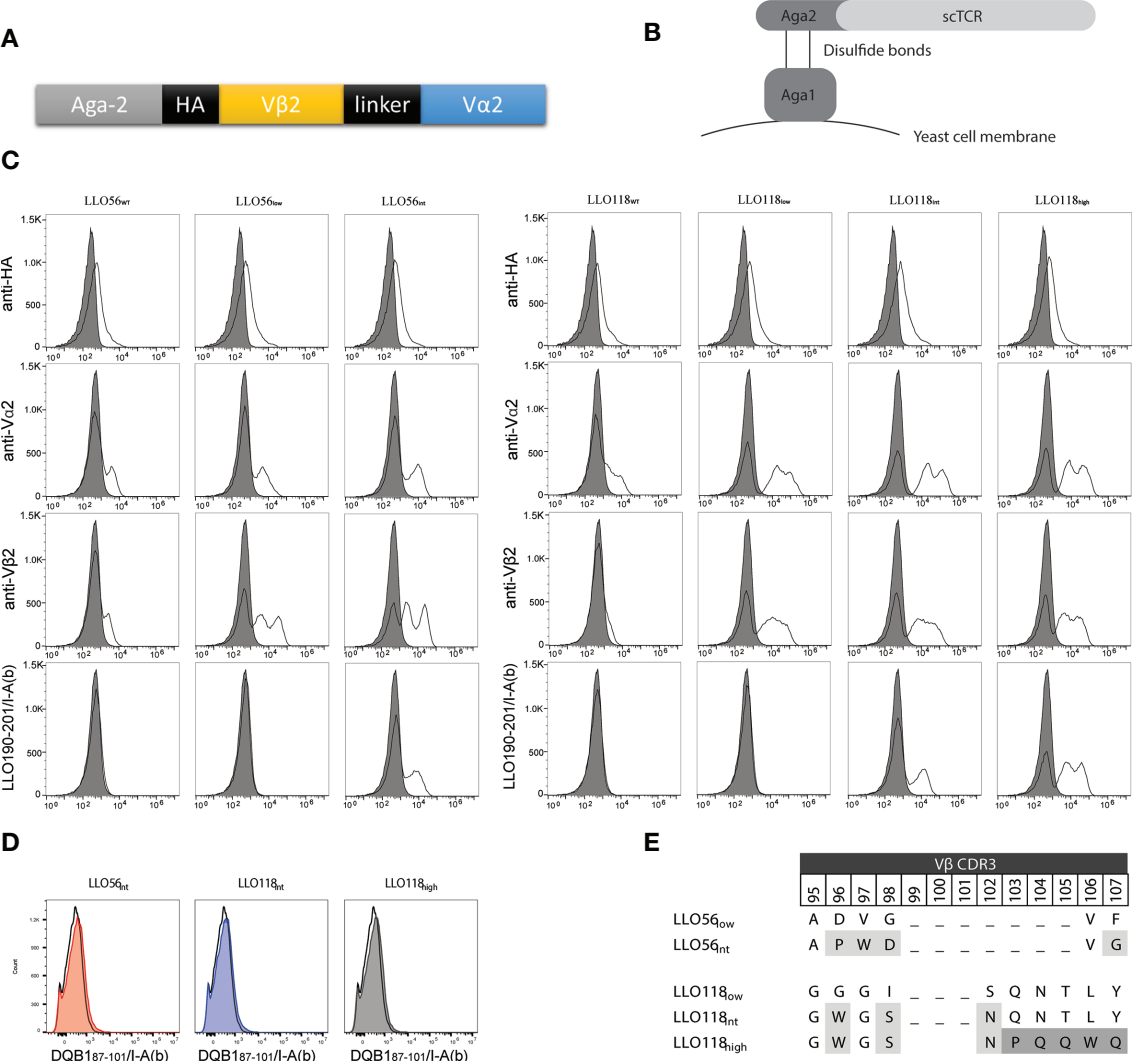
LLO118 and LLO56 stability and affinity maturation by yeast display. **(A)** Schematic of single-chain TCR (scTCR) which includes TCR α and β variable domains (Vα and Vβ) spliced from TCR constant domains and connected with a 13 amino acid linker. Aga-2 is the yeast mating protein that displays the scTCR on yeast cell membrane. Each construct contains an HA tag for antibody detection. **(B)** Schematic of a scTCR displayed on the cell surface *via* yeast display. The yeast mating protein Aga1 binds to the Aga2-scTCR fusion protein and enables the display of these proteins on the cell surface. Large scTCR libraries can be generated, stained with antibodies or peptide-MHC tetramers, and yeast clones containing scTCRs with improved stability or affinity characteristics can be identified and selected. **(C)** Wild type LLO118 and LLO56 were engineered for improved stability and higher affinity by yeast surface display. Clones with stability mutations were selected for using monoclonal anti-Vα or anti-Vβ antibodies. The surface displayed constructs were then selected for improved affinity using the peptide MHC tetramer LLO_190-201_/I-A^b^. Staining of wild type clones LLO56_WT_ and LLO118_WT_ (first and fourth columns), stability clones LLO56_low_ and LLO118_low_ (second and fifth columns), intermediate affinity clones LLO56_int_ and LLO118_int_ (third and sixth columns) and high affinity clone LLO118_high_ (seventh column) is shown. Stains include antibodies against the HA epitope (first row), Vα2 (second row), and Vβ2 (third row), or LLO_190-201_/I-A^b^ pMHCII tetramer (fourth row). Gray-filled histogram represents cells-only control. Histograms are representative of n >3 experiments. **(D)** Affinity clones were incubated with saturating amounts of non-target tetramer (DQB1_87-101_/I-A^b^). Histograms compare cells only (black clear) with affinity clones (colored, shaded). **(E)** Vβ2 CDR3 mutations that confer increases in affinity. CDR3β regions are hypervariable; therefore, gaps mark the length of other known Vβ2 CDR3β regions. First round of affinity selection (light gray) for all affinity clones while second round of affinity selection (dark gray) applies only to LLO118_high_.

Murine transgenic helper T cells LLO56 and LLO118 bear TCRs, which recognize the same naturally occurring *Listeria monocytogenes* peptide (LLO_190-205_) presented on MHCII (I-A^b^). The LLO56 and LLO118 TCR bind cognate pMHC with similar affinity (27.4 µM and 28.3 µM, respectively), yet have unique primary and secondary responses to TCR stimulus (summarized in **Supplemental Table S1**) (41, 42). LLO56 and LLO118 differ from each other by 15 amino acids located in the complementarity determining regions (CDR) CDR3β (amino acids 96–108, 111, 116, 118), CDR2β (aa52), and CDR3α (aa93) regions (**Supplemental Figure S1**). To further elucidate the effects of TCR-pMHCII affinity on CD4^+^ T cell activation, the variable regions of LLO56 and LLO118 (**Figure 1A**) were used as templates for generating a panel of single-chain TCRs (scTCRs) with low (wild type), intermediate, and high affinities. scTCR libraries generated by random mutagenesis and expressed *via* yeast surface display (**Figure 1B**) were selected for protein folding stability through magnetic column sorting (**Figure 1C**). *scTCR expression levels vary according to yeast cell cycle stage and can result in multiple peaks. The left peak is the non-displaying fraction and there can be intermediate and high displaying yeast (*61*)*. Vβ2 stability mutations were conserved between constructs while Vα2 stability mutations clustered in known stability hotspots (**Supplemental Figure S1**). To generate affinity mutants, five amino acids in the stability mutants LLO56_low_ and LLO118_low_ CDR3β region were mutated by site directed mutagenesis and selected for improved binding affinity for LLO_190-201_/I-A^b^ tetramers by magnetic column sorting. Increases in scTCR affinity cannot be explained by increases in scTCR expression, as HA and TCRα and TCRβ antibody binding remained the same across each experiment (**Figure 1B**). Additionally, none of the isolated stability or affinity mutants bound significantly to a non-target peptide tetramer (DQB1_87-101_/I-A^b^), indicating that the increase in tetramer binding is due to peptide-specific binding and not increased affinity for I-A^b^ alone (**Figure 1D**). Affinity mutant LLO56_int_ with four CDR3β mutations (**Figure 1E**) bound LLO_190-201_/I-A^b^ 1.5 log better than stability mutant LLO56_low_ (**Figure 1C**). Affinity mutant LLO118_high_ bound to the LLO_190-201_/I-A^b^ tetramer 1.0-log better than affinity mutant LLO118_int_ and 2.5-log better than stability mutant LLO118_low_ (**Figure 1C**). LLO118_int_ had three CDR3β mutations and LLO118A_high_ had five additional CDR3β mutations (**Figure 1E**). While LLO56_int_ and LLO118_high_ mutants were used as templates for mutant libraries of the complementary determining region 3 of the α chain (CDR3α), no further clones with increased-affinity for LLO_190-201_/I-A^b^ tetramer were isolated, suggesting that CDR3β is primarily responsible for LLO_190-201_ peptide interactions for these specific TCRs. It is important to note that the stability clones initially isolated from yeast libraries relied on a frameshift mutation at the stop codon that added 19-amino acids from the yeast expression vector to the carboxy end of Vα2 (RSDNNSVDVTKSTLFPPYF). While LLO56_low_ and LLO56_int_ successfully retained stability and affinity gains without the 19 amino acids, several attempts to create new LLO118 stability and affinity clones without the additional amino acids were unsuccessful. Therefore, the stabilizing 19 amino acids were maintained for LLO118 clones. Other studies have utilized TCR formats that express constant domains in order to maintain scTCR folding while adding additional affinity mutations to the TCRs, therefore this observation was not unexpected (62–64).

**Figure 2:**
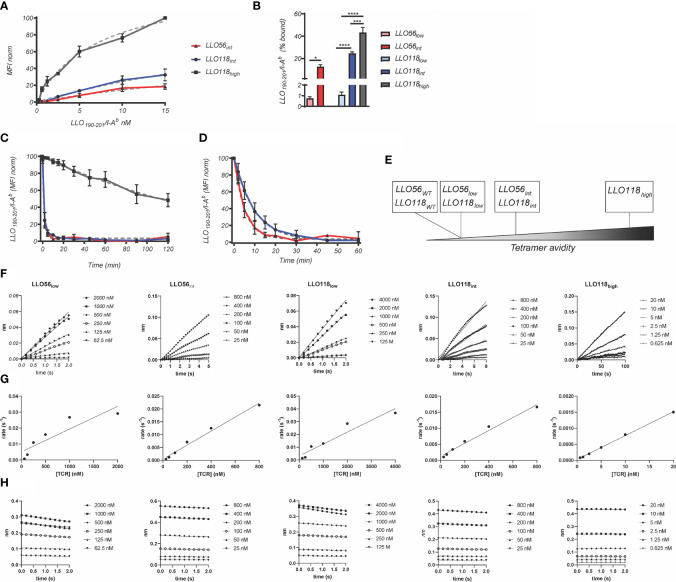
Avidity and affinity measurements of scTCRs. **(A)** To estimate avidity K_D_, affinity clones presented by yeast were incubated with various concentrations of LLO_190-201_/I-A^b^ tetramer (1.52–50 nM) and 50,000 events were collected *via* flow cytometry. Grey dotted lines represent non-linear, one site-specific binding analysis of tetramer binding measurements used to estimate K_D_ for LLO56_int_ (red), LLO118_int_ (blue), and LLO118_high_ (dark grey) (n = 3 experiments). **(B)** Percent of cells bound by tetramer for each affinity clone. LLO56_low_ had significantly lower binding than LLO56_int_ (0.75 ± 0.3% to 12.6 ± 3.4%, p = 0.0193) while LLO118_low_ (1.1 ± 0.5%) was significantly lower than LLO118_int_ (24.5 ± 2.9%) and LLO118_high_ (43.3 ± 8.9%) (p < 0.0001 and p < 0.0001, respectively) and LLO118_int_ was significantly lower than LLO118_high_ (p = 0.0004). To determine *t_1/2_*, yeast displayed affinity clones were incubated with **(C)** 1 M and **(D)** 0.1 M LLO_190-201_/I-A^b^ tetramer. Following an initial measurement, 1.0 µg/ml anti-mouse MHC class II (I-A, I-E) monoclonal antibody was added and tetramer binding measured at each time point by flow cytometry. *t_1/2_* was estimated as the time it took to reach 50% MFI modeled by non-linear dissociation one phase exponential decay (dotted light grey lines) (n = 3 experiments). **(E)** A graphical depiction of range of affinity clones. To estimate TCR affinity, scTCRs were secreted by *E*. *coli*, chemically refolded, and incubated with streptavidin sensors loaded with biotinylated LLO_190-201_/I-A^b^ monomer using BLI. No binding was observed between scTCRs and streptavidin biosensors in the absence of LLO_190-201_/I-A^b^ loading (n = 3–6 independent measurements). **(F)** k_obs_ is the linear slope of nm vs time. **(G)** k_assoc_ is the linear slope of k_obs_ vs scTCR concentration. **(H)** k_diss_ is the exponential slope of dissociation nm vs time. *<0.05, ***<0.001, ****<0.0001.

The multivalent binding avidity of each clone was determined by LLO_190-201_/I-A^b^ tetramer titration (150 pM–15.00 nM) of scTCR expressed on yeast (**Figure 2A**). Avidities ranged from the highest clone LLO118_high_ (7.30 nM), to intermediate avidity clones LLO56_int_ (39.20 nM) and LLO118_int_ (44.80 nM) (**Figures 2A, B** and **Table 1**). Stability clones LLO56_low_ and LLO118_low_ were excluded from these analyses because binding was undetectable even at the highest concentrations of LLO_190-201_/I-A^b^ tetramer (**Figure 2B**). Tetramer decay analysis of clones displayed on yeast determined that the multivalent half-life for LLO118_high_ (*t_½_* = 165 min, r^2^ = 0.76) was 165-times longer than LLO118_int_ and LLO56_int_ (*t_½_* = ~1 min each, r^2^ = 0.97 each) suggesting that the increased avidity of LLO118_high_ is predominantly due to a lengthened off-rate (**Figure 2C**). A second round of tetramer decay with lower levels of MHC inhibiting-antibody better resolved the half-lives of LLO118_int_ (*t_½_* = 6.7 mins, r^2^ = 0.97) and LLO56_int_ (*t_½_* = 3.5 mins, r^2^ = 0.98) (**Figure 2D**), indicating that LLO118_int_ has a longer dissociation rate than LLO56_int_. The resulting panel of TCRs provides a range of tetramer avidities ranging from high to low (**Figure 2E**).

**Table 1:** Comparison between scTCR clones avidity (Tetramer) and affinity K_d_ (Bio-layer Interferometry).

	Tetramer (Avidity)				Bio-layer Interferometry (Affinity)			
	t_1/2_ (m)	r^2^	K_D_	r^2^	k_assoc_ (M^-1^s^-1^)	k_dissoc_ (s^-1^)	t_1/2_ (s)	K_D_
*LLO56_low_*					18300 ± 7000	0.053 ± 0.020	14.5 ± 5.7	2.9 ± 0.7 µM
*LLO56_int_*	1	0.97	39.2 ± 46.7 nM	0.82	27600 ± 3000	0.018 ± 0.0007	422.0 ± 163.1	66.2 ± 39.8 nM
*LLO118_low_*					10200 ± 1500	0.043 ± 0.004	16.1 ± 1.3	4.3 ± 0.7 µM
*LLO118_int_*	1	0.97	44.8 ± 52.3 nM	0.96	20000 ± 1400	0.025 ± 0.007	28.8 ± 7.4	1.3 ± 0.3 µM
*LLO118_high_*	165	0.76	7.33 ± 1.37 nM	0.87	73000 ± 8600	0.002 ± 0.0007	460.0 ± 176.8	20.0 +/- 13.9 nM

While tetramer avidity measurements may be more physiologically relevant as multiple TCR-pMHCs interact simultaneously during T cell activation, TCR-pMHC affinity measurements provide a standard measurement to compare between TCR systems. Therefore, TCR:pMHC affinity was measured by quantifying the interaction of monomeric refolded scTCR with monomeric LLO_190-201_/I-A^b^ *via* bio-layer interferometry. Due to non-specific binding at the later stages of the association and dissociation steps, the K_D_ was calculated manually by extracting the data from the early measurements; k_obs_ slopes ((**Figure 2F**) were plotted against scTCR concentration (**Figure 2G**) and k_assoc_ estimated from the slope. k_diss_ is the slope of dissociation graphs (**Figure 2H**). K_D_ was determined by dividing k_diss_/k_assoc_. LLO118_high_ (20.0 ± 13.9 nM) K_D_ was 215-fold higher than LLO118_low_ (4.3 ± 0.7 µM) (**Table 1**). Intriguingly, while LLO118_int_ and LLO56_int_ avidity measurements were similar, their affinity measurements were markedly different (20-fold). LLO118_int_ (1.3 ± 0.3 µM) was only 3-fold higher affinity than LLO118_low_ and LLO56_int_ (66.2 ± 39.8 nM) was 43-fold higher than LLO56_low_ (3.8 ± 1.3 µM) (**Table 1**).

### Construct Format Impacts Surface Expression and pMHCII-Affinity Independently

**Figure 3:**
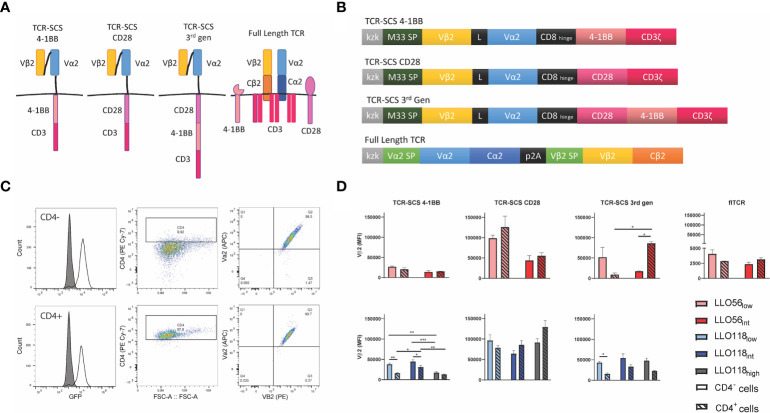
TCR-SCS and flTCRs are stably expressed in CD4^-^ and CD4^+^ T cell hybridomas. **(A)** Diagrams depicting the flTCR formats and three TCR-SCS formats in the cell membrane. All TCR-SCS constructs have signaling coreceptor CD3 in addition to the signaling domains of coreceptors CD28 and/or 4-1BB. **(B)** TCR constructs are produced from a bicistronic IRES-GFP vector and GFP expression mirrors TCR construct expression in cell lines. In addition to the P2A cleavage domain, TCR-SCS formats rely on TCR M33 signal peptide (67), whereas the flTCR has dedicated signal peptides for α2 and β2 to increase localization of both chains to the surface. The CD8 hinge acts as an intermembrane domain. **(C)** An example of TCR-SCS expression in CD4^-^ (top) and CD4^+^ (bottom) T cell hybridoma lines after magnetic column sorting with anti-Vβ2 monoclonal antibodies (LLO118_high_ TCR-SCS CD28). Initial retroviral transfections ranged from 11 to 85% efficiency. Grey peaks in GFP histograms represent a GFP^-^ cells-only control. GFP^+^ cells were gated for CD4 expression and Vα2 and Vβ2 expression. Representative of n = 3 measurements of 20,000 cells *via* flow cytometry. **(D)** Mean fluorescent intensity (MFI) of Vβ2 was used as a proxy for stable expression of constructs. Constructs were expressed in CD4^-^ (solid bars) and CD4^+^ (hatched bars) T cell hybridomas. Representative of n = 3 measurements of 20,000 cells *via* flow cytometry. *<0.05, **<0.01, ***<0.001.

To quantitatively assess the effects of TCR-pMHC affinity, CD4, and construct format on helper T cell activation, TCR constructs were retrovirally transduced into murine T cell hybridomas, 58^-/-^ CD4^-^ (CD4^-^) and 58^-/-^ CD4^+^ (CD4^+^), which do not express endogenous TCRs. 58^-/-^ T cell hybridoma cell lines have been a useful cell line for examining TCR kinetics and IL-2 production prior to primary cell line observations (47, 49, 65, 66). LLO56_low_ and LLO56_int_ were placed in the three TCR-SCSs formats, and LLO56_WT_ and LLO56_int_ were placed in flTCR constructs without stability mutations (**Figures 3A, B**, **Supplemental Figures S2**–**S5**). Because of the necessity of the additional 19 amino acids, LO118_low_, LLO118_int_, and LLO118_high_ affinity changes were not transferred to flTCR constructs. LLO118_low_, LLO118_int_, and LLO118_high_ were placed in three TCR-SCSs formats based on commonly used second and third generation chimeric antigen receptor (CAR) formats (**Figures 3A, B**, **Supplemental Figures S2**–**S4**). The transduced cell lines were sorted with anti-Vβ2 antibodies *via* magnetic column selection for >85% GFP^+^ and TCR expression (**Figure 3C**).

TCR stable surface expression varied by individual constructs. As assessed by Vβ2 expression, flTCR constructs were less stably expressed than all TCR-SCS constructs perhaps due to CD3 subunit availability, while TCR-SCS CD28 constructs were the most stably expressed format for both LLO56 and LLO118 constructs (**Figure 3D**). While most construct expression was equitable between CD4^-^ and CD4^+^ cell lines, TCR-SCS 4-1BB constructs had the most expression variability between constructs as CD4 expression destabilized LLO118_low_ and LLO118_int_ TCR-SCS 4-1BB expression (p = 0.0018 and p = 0.0429, respectively) (**Figure 3D**). CD4^-^ LLO118_high_ 4-1BB constructs were less stable than CD4^-^ LLO118_low_ 4-1BB and LLO118_int_ 4-1BB (p = 0.0030 and p = 0.0002, respectively) (**Figure 3D**). CD4^+^ LLO118_high_ 4-1BB was also less stable than either LLO118_low_ or LLO118_int_ (p = 0.0285 and p = 0.0493) (**Figure 3D**). Overall CD4 expression did not significantly destabilize 3^rd^ gen constructs except LLO118_low_ (p = 0.0401) and LLO56_int_ where CD4 stabilized VB2 expression (p = 0.0109) (**Figure 3D**). GFP expression does not correlate with expression differences of the constructs for each format between CD4^-^ and CD4^+^ expression nor expression differences between TCR (**Supplemental Figures S6**).

**Figure 4:**
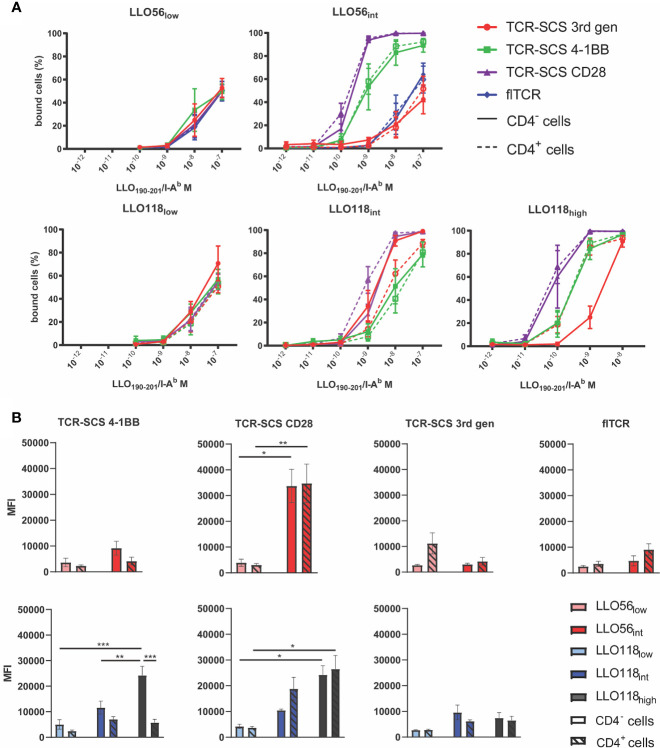
TCR-pMHCII avidity is affected by construct format. **(A)** flTCR or TCR-SCS expressing cell lines were incubated with varying amounts of LLO_190-201_/I-A^b^ tetramer (10^-7^ M to 10^-12^ M) at room temperature for 2 h. Each TCR-format pair expressed in CD4^-^ cell lines (solid lines) and CD4^+^ cell lines (dotted lines) have similar affinities, whereas each unique construct format alters avidity of a single TCR. Representative of three independent measurements of 20,000 cells *via* flow cytometry. **(B)** Tetramer MFI measurements of 10^-8^ M separated by TCR and format where CD4^-^ cell lines (solid bars) and CD4^+^ cell lines (hashed bars) are paired. *<0.05, **<0.01, ***<0.001.

Tetramer titrations were used to approximate the avidity of each flTCR or TCR-SCS construct. Intriguingly, the intracellular format strongly influenced the avidity of each intermediate and high affinity TCR construct (**Figure 4A**). There is no clear link across all clones between stable Vβ2 expression and construct avidity, although the most stable constructs—TCR-SCS CD28—did have the highest apparent avidity (LLO56_int_ and LLO118_high_) (**Figure 4A**). Overall, CD4 expression (dotted lines) did not affect the avidity of the constructs, excepting LLO118_int_ and LLO118_high_ 3^rd^ gen constructs where CD4 lessened and heightened avidity, respectively (**Figure 4A**). The MFI measured for each clone at 10^-8^ M (a non-saturated concentration) were used to compare avidity differences between affinity clones. LLO56 4-1BB, 3^rd^ gen and flTCR constructs had no significant differences between LLO56_low_ and LLO56_int_ (**Figure 4B**). This may be due to the small affinity differences between LLO56_low_ and LLO56_int_ as measured in tetramer and bio-layer interferometry assays. However, LLO118 3^rd^ gen constructs also did not show affinity-dependent avidity changes, thus intracellular signaling domains may also affect the avidity of extracellular scTCRs. There were significant avidity differences for LLO118 4-1BB clones; CD4^-^ LLO118_high_ 4-1BB had significantly better avidity than its cognate CD4^+^ pairing (p = 0.0004), and was also significantly higher than CD4^-^ LLO118_low_ and LLO118_int_ 4-1BB (p = 0.0003 and p = 0.0092, respectively) (**Figure 4B**). Additionally, TCR-SCS CD28 constructs for both LLO56 and LLO118, which are the most stably expressed constructs (**Figure 3D**), showed increased MFI by increasing TCR affinity (**Figure 4B**). LLO56_int_ CD28 had significantly greater avidity than LLO56_low_ CD28 (CD4^-^ p = 0.0122 and CD4^+^ p= 0.0086), as did LLO118_high_ CD28 compared to LLO118_low_ (CD4^-^ p = 0.0129 and CD4^+^ p= 0.0113) (**Figure 4B**). IL-2 production is not correlated with GFP intensity (**Supplemental Figure S6**). Taken together, while there is no systematic correlation, this data suggests that construct stability may influence avidity measurements, as CD28 clones had the highest stability and avidity, and confirms that generally, CD4 does not affect perceived avidity.

### CD4 Inhibits High Affinity TCR IL-2 Production

**Figure 5:**
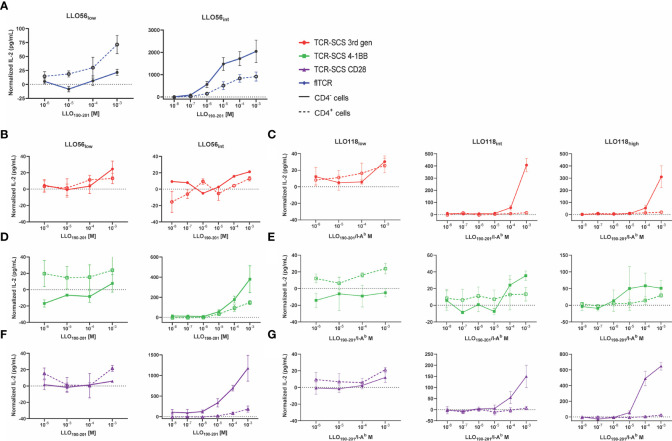
CD4 inhibits IL-2 production of intermediate and high affinity TCRs. CD4^-^ (solid lines) and CD4^+^ (dotted lines) T cell hybridoma cell lines were incubated with various concentrations of LLO_190-201_ peptide (10^-8^ M to 10^-3^ M) presented by BL6/C57 splenocytes for 24 h. IL-2 production was measured by ELISA. Each sample was normalized by subtracting baseline IL-2 production from T cell hybridoma/splenocytes controls incubated without peptide. **(A)** LLO56 flTCRs, **(B)** LLO56 3^rd^ gen TCR-SCSs, **(C)** LLO118 3^rd^ gen TCR-SCSs, **(D)** LLO56 4-1BB TCR-SCSs, **(E)** LLO118 4-1BB TCR-SCSs, **(F)** LLO56 CD28 TCR-SCSs, and **(G)** LLO118 CD28 TCR-SCSs. ELISAs run n = 3 times.

To assess the effects of TCR-pMHCII affinity, CD4 expression, and format on T cell activation we measured IL-2 expression in response to increasing agonist peptide concentrations. As anticipated, LLO56_low_ flTCR IL-2 production improved with CD4 expression, but CD4 expression unexpectedly reduced IL-2 production for LLO56_int_ flTCR (**Figure 5A**). Despite the inconsistent role of CD4, flTCRs produced significantly more IL-2 at all affinity levels (**Figure 5A**) and were at least 1-log fold more sensitive to peptide than all TCR-SCSs (**Figures 5B–D**). IL-2 production for CD4^-^ clones rose with increased TCR affinity for most constructs except 3^rd^ gen constructs; LLO56_int_ 3^rd^ gen failed to produce more cytokines than LLO56_low_ 3^rd^ gen (**Figure 5B**) and LLO118_high_ 3rd gen that produced less IL-2 than LLO118_int_ 3^rd^ gen (**Figure 5C**). This pattern of uneven gains across affinity and base TCR was also observed for 4-1BB constructs (**Figures 5D, E**); while LLO56 4-1BB did see gains across affinity (**Figure 5D**), LLO118 4-1BB constructs had limited affinity gains across the affinity gradient (**Figure 5E**). CD4^-^ LLO56_int_ CD28 and CD4^-^ LL0118_high_ CD28 produced more IL-2 than other TCR-SCS constructs which suggested that their heightened stable expression may promote IL-2 production (**Figures 5F, G**). As noted in low affinity scTCR clones (LLO56_low_ 3^rd^ gen and CD28, and LLO118_low_ 3^rd^ gen) (**Figures 5B, C, F**), CD4^+^ and CD4^-^ clones may also respond uniquely across antigenic concentrations, however this is likely an artifact due to variability or the limits of detection.

While CD4 promoted the activation of all low affinity clones, it unexpectedly suppressed IL-2 production for all intermediate and high affinity constructs (**Figure 5**). The magnitude of IL-2 suppression is greatly dependent on whether the construct was a flTCR or TCR-SCS construct. For example, while LLO56_low_ flTCR IL-2 production was assisted by CD4 expression, LLO56_int_ flTCR IL-2 production was reduced 2.2-fold (p = 0.0707) at 10^-3^ M peptide stimulation (**Supplemental Figure S7**). In contrast, only one TCR-SCS had such a mild IL-2 reduction. CD4^+^ LL056_int_ 4-1BB IL-2 production was reduced by 2.5-fold (p = 0.1551) (**Supplemental Figure S7**). The IL-2 production for the other intermediate and high affinity TCR-SCS constructs was intermediately reduced for LLO56_int_ CD28 (6.4-fold, p = 0.0104), and severely reduced for LLO118_int_ 3^rd^ gen (28.1-fold, p = 0.0004), LLO118_int_ CD28 (21.2-fold, p = 0.0400), LLO118_high_ 3^rd^ gen (16.5-fold, p = 0.0051), and LLO118_high_ CD28 (25.9-fold, p < 0.0001) (**Supplemental Figure S7**). Peptide sensitivity, defined as the lowest concentration where IL-2 response exceeds baseline IL-2 production, was equitable between CD4^-^ and CD4^+^ for constructs LLO56_int_ flTCR, LLO56 3^rd^ gen, and LLO56_int_ 4-1BB (**Figures 5A, D**, **Table 2**), but delayed 1-log fold for LL056_int_ CD28 and at least 2-log fold for all LLO118_int_ and LLO118_high_ constructs (**Figures 5C, E–G**, **Table 2**). This suggests that CD4 reduced peptide sensitivity for most TCR-SCS constructs, possibly in a TCR-dependent manner.

**Table 2:** Approximated peptide sensitivity for responding clones.

Peptide sensitivity						
	*LLO56int*		*LLO118int*		*LLO118high*	
	CD4-	CD4+	CD4-	CD4+	CD4-	CD4+
*flTCR*	1.00E-06	1.00E-06				
*3rd gen*	1.00E-04	1.00E-04	1.00E-04	ND	1.00E-04	ND
*4-1BB*	1.00E-05	1.00E-05	1.00E-04	ND	1.00E-06	1.00E-04
*CD28*	1.00E-05	1.00E-04	1.00E-04	ND	1.00E-05	ND

CD4 expression inhibited the production of IL-2 for most clones (marked in light red).

### Lck Sequestration by CD4 Inhibits Some TCR IL-2 Production

**Figure 6:**
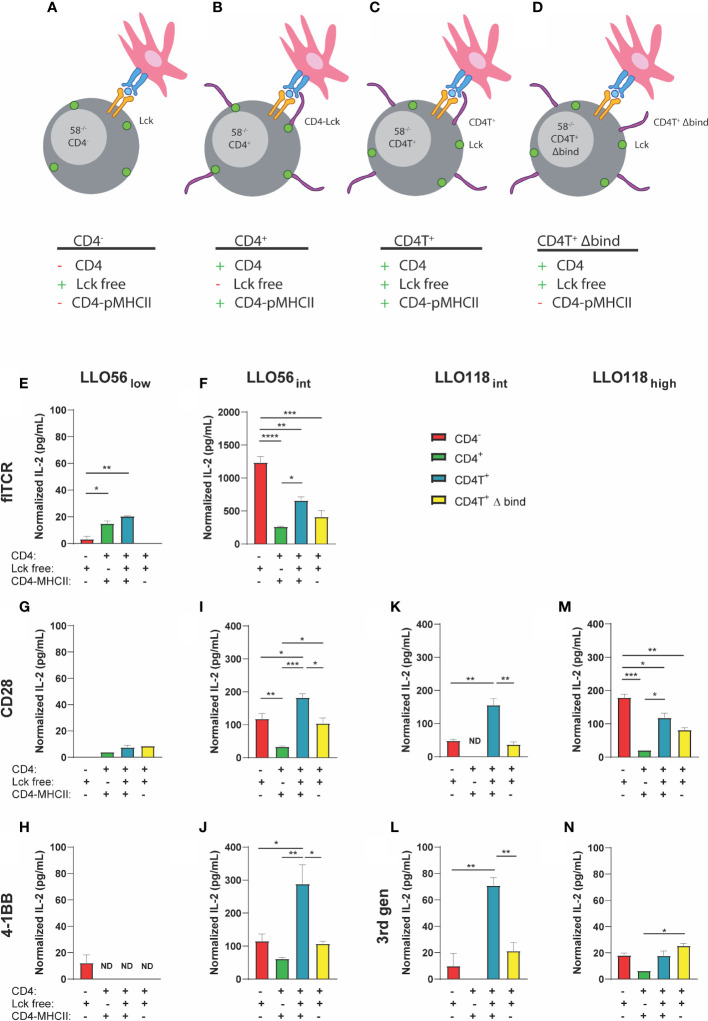
IL-2 production of intermediate and high affinity clones is influenced by Lck sequestration, CD4-MHCII interaction, and CD4 presence. **(A)** CD4^-^ has no CD4 and Lck is spread ubiquitously along the inner membrane. **(B)** CD4^+^: wild type CD4 interacts with MHCII and the majority of Lck is sequestered to the cytoplasmic tail of CD4. **(C)** CD4T^+^: mutant CD4T interacts with MHCII but does not sequester Lck which is spread ubiquitously along the inner membrane. **(D)** CD4T^+^ Δ bind: mutant CD4T Δ bind does not bind to MHCII nor Lck (Lck is not sequestered to the cytoplasmic tail of CD4). flTCR and SCS-TCR constructs expressed in various T cell hybridoma clones [CD4^-^ (red), CD4^+^ (green), CD4T^+^ (light blue), CD4T^+^ Δ bind (yellow)] were incubated with 10^-3^ M LLO_190-205_ presented on Bl6.C57 splenocytes for 24 h. IL-2 production was measured by ELISA and normalized by subtracting IL-2 production without peptide for each clone-APC pair. Histogram order: LLO56_low_ **(E)** and LLO56_int_ **(F)** flTCRs; LLO56_low_ **(G)**, LLO56_int_ **(H)**, LLO118_int_ **(I)**, and LLO118_high_ **(J)** CD28 TCR-SCS; LLO56_low_ **(K)** and LLO56_int_ **(L)** 4-1BB SCS-TCR; LLO118_int_ **(M)**, and LLO118_high_ **(N)** 3^rd^ gen TCR-SCS. p-values were determined by multiple comparison one-way ANOVA for each graph are from left to right LLO56_int_ 4-1BB CD4^-^ to CD4T^+^ (p = 0.0176), CD4^+^ to CD4T^+^ (p = 0.0036), and CD4T^+^ to CD4T^+^ Δ bind (p = 0.0138); LLO56_int_ CD28 CD4^-^ to CD4^+^ (p = 0.0054), CD4^-^ to CD4T^+^ (p = 0.0232), CD4^+^ to CD4T^+^ (p = 0.0002), CD4^+^ to CD4T^+^ Δ bind (p = 0.0245), and CD4T^+^ to CD4T^+^ Δ bind (p = 0.0153); LLO118_int_ 3^rd^ gen CD4^-^ to CD4T^+^ (p = 0.0027), and CD4T^+^ to CD4T^+^ Δ bind (p = 0.0077); LLO118_int_ CD28 CD4^-^ to CD4T^+^ (p = 0.0018), and CD4T^+^ to CD4T^+^ Δ bind (p = 0.0010); LLO118_high_ 3^rd^ gen CD4^+^ to CD4T^+^ Δ bind (p = 0.0366); and LLO118_high_ CD28 CD4^-^ to CD4^+^ (p = 0.0010), CD4^-^ to CD4T^+^ (p = 0.0221), CD4T^-^ to CD4T^+^ Δ bind (p = 0.0021), and CD4^+^ to CD4T^+^ (p = 0.0117). *<0.05, **<0.01, ***<0.001, ****<0.0001.

Lck is an early proximal signaling kinase that colocalizes to the cytoplasmic domain of CD4 (68, 69). If Lck is poorly recruited to the TCR-pMHCII synapse, then T cell activation may be diminished (49). We hypothesized that our high affinity clones may poorly recruit CD4-Lck to the immunological synapse, decreasing activation, and therefore reducing IL-2 production as observed in CD4^+^ intermediate and high affinity clones. To parse out the potential contributions of CD4-Lck sequestration, CD4-MHCII interactions, and any CD4-dependent inhibition, we expressed a selection of our flTCR and TCR-SCS clones in four 58^-/-^ T cell hybridoma lines (49, 60). LLO56 TCR-SCS 3^rd^ gen and LLO118 TCR SCS 4-1BB clones were dropped due to their poor performance in the first IL-2 tests. The 58^-/-^ CD4^-^ T cell hybridoma cell line (CD4^-^) lack CD4, which allows Lck to interact freely with the TCR-pMHCII complex and nullifies CD4-MHCII interactions (**Figure 6A**). The 58^-/-^ CD4^+^ T cell hybridoma cell line (CD4^+^) has wild type CD4 which sequesters Lck to its cytoplasmic tail and binds to MHCII (**Figure 6B**). 58^-/-^ CD4T^+^ T cell hybridoma line (CD4T^+^) is truncated C-terminally (maintains amino acids 1-421) which allow Lck to colocalize but not bind to CD4 while CD4 still binds to MHCII (**Figure 6C**) (60, 70). CD4T expressed in both T cells and hybridomas has been documented in many sources to not bind Lck (60, 70, 71) and is suggested to produce IL-2 in a Lck-independent manner (60, 70, 71). Previous work has also demonstrated in cells that, while CD4TΔbind does not bind MHCII, it still contains the cytoplasmic tail necessary for binding Lck (72, 73).Finally, 58^-/-^ CD4T^+^ Δbind (CD4T^+^ Δbind) frees Lck and is mutated to prevent CD4 binding to MHCII by altering residues 68–73 from KGVLIR to DGDSDS (**Figure 6D**) (60).

CD4T^+^ and CD4T^+^ Δbind constructs were retrovirally transduced into existing CD4^-^ T cell hybridomas containing TCR-SCS or flTCR constructs and the clones were sorted for GFP, TCR, and CD4 expression by flow sorting. CD4^-^ hybridomas did not express CD4, and there was consistent CD4 expression between the various CD4^+^ clones (**Supplemental Figure S8**) while GFP levels varied (**Supplemental Figure S9A**). TCR surface expression was consistent across cell lines for most TCR constructs, except LLO56_low_ and LLO56_int_ CD28 clones which were most stably expressed in CD4^+^ cells (p < 0.0001 and p < 0.0001, respectively) (**Supplemental Figure S9B**). Similarly, avidity measured by tetramer was mainly consistent between clones except for LLO56_low_ 4-1BB where CD4^-^ had higher avidity than all CD4^+^ clones (p = 0.0014), and LLO118_int_ CD28 where CD4^+^ clone had the highest avidity (p = 0.0063) (**Supplemental Figure S9C**).

As expected, LLO56_low_ flTCR IL-2 production was promoted by the presence of CD4-MHCII interactions (CD4^+^ p = 0.0105 and CD4T^+^ p = 0.0014) and the absence of CD4-MHCII interaction in the CD4^-^ and CD4T^+^ Δbind abrogated LLO56_low_ flTCR IL-2 production (**Figure 6E**). CD4 Lck-sequestration did not affect LLO56_low_ flTCR IL-2 production since the CD4^+^ and CD4T^+^ clones responded the similarly to antigen (**Figure 6E**). In contrast, intermediate affinity LLO56_int_ flTCR IL-2 production is inhibited by CD4 Lck-sequestration, as CD4^+^ produced significantly less IL-2 than CD4T^+^ (p = 0.0162) (**Figure 6F**). However, the more striking phenotype is LLO56_int_ flTCR CD4-dependent inhibition, as IL-2 production is significantly reduced by the presence of CD4 in any form (**Figure 6F**). CD4^-^ LLO56_int_ flTCR cells produced significantly more IL-2 than any clone expressing CD4 (CD4^+^ p < 0.0001, CD4T^+^ p = 0.0019, and CD4T^+^ Δbind p = 0.0002) (**Figure 6F**). Furthermore, CD4-MHCII interaction was not a significant contributor to intermediate affinity flTCR IL-2 production as there was no significant change in IL-2 production between the CD4T^+^ and CD4T^+^ Δbind clones (**Figure 6F**).

Low affinity TCR-SCS clones LLO56_low_ CD28 (**Figure 6G**) and LLO56_low_ 4-1BB (**Figure 6H**) were low IL-2 producers and the role of CD4 was conflicting as all CD4 iterations inhibited IL-2 production for 4-1BB but promoted IL-2 production for CD28 whether or not CD4 binds to MHCII. It was also difficult to draw conclusions about Lck-sequestration for low affinity TCR-SCS constructs due to low levels of IL2 production. Intermediate TCR-SCS clones LLO56_int_ CD28 (**Figure 6I**), LLO56_int_ 4-1BB (**Figure 6J**), LLO118_int_ CD28 (**Figure 6K**), and LLO118_int_ 3^rd^ gen (**Figure 6L**) had a unique ubiquitous phenotype comparable to the phenotype described for intermediate affinity flTCR clones. IL-2 production was most reduced when CD4 sequestered Lck in the CD4^+^ clones (**Figures 6I–L**). However, intermediate TCR-SCS CD4T^+^ constructs produced the most IL-2, indicating unrestricted Lck promotes the greatest T cell activation (**Figures 6I–L**). CD4T^+^ Δbind compared to CD4T^+^ significantly reduced intermediate TCR-SCS construct IL-2 production to CD4^-^ levels suggesting that CD4-MHCII binding supports IL-2 production for intermediate TCR-SCS affinity (**Figures 6I–L**). Noticeably, high affinity LLO118 TCR-SCSs followed the same inhibition patterns seen for LLO56_int_ flTCR where inhibition by Lck sequestration and CD4 presence was not significantly affected by MHCII-CD4 binding (**Figures 6M, N**). Taken together, these data indicate that flTCRs and TCR-SCS have independent affinity thresholds for the inhibitory effects of Lck-sequestration and CD4-dependent inhibition, and the activation promoting effects of CD4-MHCII interactions (summarized in **Table 3**). Thus, IL-2 inhibition is affected by CD4-Lck sequestration, CD4-pMHCII interaction, and by a CD4-dependent mechanism in an affinity- and format-dependent manner.

**Table 3:** Summary of **Figures 6**.

		low			int			high		
		CD4	Lck-free	CD4-MHCII	CD4	Lck-free	CD4-MHCII	CD4	Lck-free	CD4-MHCII
LLO56	flTCR	NA	NA	+	**-**	**+**	**NA**			
	4-1BB	–	+	NA	NA	+	+			
	CD28	+	NA	NA	NA	+	+			
LLO118	CD28				NA	+	+	**-**	**+**	**NA**
	3rd gen				NA	+	+	**NA**	**+**	**NA**

IL-2 production data interpretation broken down into base TCR (LLO56 or LLO118), construct, and TCR-pMHCII affinity. “-” indicates that the condition inhibits or does not promote IL-2 production, “NA” indicates that effects on IL-2 production were “not appreciable”, and “+” indicates that the condition promotes or least does not inhibit IL-2 production. Bolded interior boxes highlight the phenotype shared by intermediate affinity flTCR and high affinity TCR-SCS clones.

## Discussion

Here, we engineered and characterized a panel of MHCII-specific TCRs with increasing pMHC affinity in order to interrogate the relationships between TCR format, TCR-pMHCII affinity, and the coreceptor CD4 on CD4^+^ T cell activation. In addition to the generation of a high affinity MHCII-dependent TCR model, we identify a CD4-dependent phenotype potentially relevant for cancer-immunotherapeutic development and show that high affinity flTCRs outperform TCR-SCS formats, that TCR-SCS format effects on T cell activation are more dependent on the TCR than the TCR-SCS format, and that CD4 can inhibit both flTCR and TCR-SCS activation in an Lck dependent and independent fashion. This study utilized 58^-/-^ T cell hybridomas as a proxy for T cell activation activity. While not as physiologically relevant as using primary T cells, this system has the advantage of enabling the survey of T cell activation characteristics for multiple constructs as we have done here and has been frequently used as a springboard for further exploration of high affinity TCRs in primary T cells (47, 65, 74, 75). The contributions of these factors were assessed using IL-2 production, which is a proxy, but not a complete indication of T cell activation. flTCRs produced more IL-2 than all TCR-SCS constructs at each affinity level and IL-2 production generally increased with rising TCR affinity for all constructs. In low affinity TCRs, CD4 enhanced IL-2 production for both flTCR and TCR-SCS formats. For intermediate or high affinity TCR clones, IL-2 production was abrogated by CD4-Lck sequestration and an unknown CD4-dependent mechanism. These effects, activation promotion by increased affinity and CD4-MHCII, or activation suppression by Lck-sequestration and CD4 itself, had unique affinity thresholds that are dependent on construct type (flTCR or TCR-SCS). Lck sequestration affected activation for all intermediate and high affinity constructs, while CD4-MHCII ceased to promote activation and CD4-dependent inhibition repressed IL-2 production at unique affinity thresholds for flTCR constructs (intermediate affinity) and TCR-SCS constructs (high affinity). It is possible that Lck sequestration or the constructs themselves, have unique and important effects on other activation markers (such as the early activation markers CD25, pLCk, pCD3ζ, pERK, CD69), inhibitory markers (such as PD1 and LAG3), tonic signaling, T cell proliferation and effector function, which should be investigated in primary T cells in the future.

The balance of free unbound Lck and coreceptor-bound Lck affects T cell developmental fate, and T cell responsiveness in the periphery. Following colocalization to the TCR, CD4 signals *via* Lck bound to its cytoplasmic tail (68, 69). Lck phosphorylates immune-receptor tyrosine-based activating motifs (ITAMs) of the CD3 subunits of the TCR complex, which then initiates other early signaling machinery of the T cell (69, 76, 77). Bound and unbound Lck signal independently and can alter T cell development and function (78, 79). During thymic selection, the intracellular coreceptor-bound or unbound state of Lck determines whether αβ TCRs are MHC-restricted or independent (78). Lck association with coreceptor proteins determines MHC restriction (78), and coreceptor-Lck binding stoichiometry is the limiting factor for signaling during selection (80). In particular, CD8, which binds Lck more preferentially than CD4, has a greater effect on TCR selection and increases CD8^+^ T cell reactivity to low affinity and self-reactive antigen compared to CD4^+^ T cells (80). In both mature CD8^+^ T cells and T cell hybridomas, free Lck has higher mobility, more activating Y394 phosphorylation, higher kinase activity, and mediated higher T cell activation compared to coreceptor-bound Lck (81). Additionally, during activation, TCR-CD3 is first phosphorylated by unbound-Lck followed by MHC-dependent CD3-CD8 interaction and the less activated coreceptor-bound Lck (82–84). CD4-bound Lck activation may be reliant on a mechanism distinct from CD4-free Lck activation, which is likely mediated by tyrosine-protein kinase Fyn and may obscure mechanism comparison (37, 85, 86). Additionally, it is also possible that CD4 may function differently in T cells expressing native TCRs or CAR cytoplasmic domains. However, despite these potential complications, CD4-Lck-dependent inhibition could occur in two fashions. First, optimal TCR affinity-mediated signaling is dependent on fine-tuning the intensity and duration of the Lck phosphorylation cascade and high affinity TCRs may have early intense Lck phosphorylation resulting in acute transient activation (87). Conversely, if CD4 is not recruited to the TCR, it could sequester Lck away from the activation complex, which prevents the activation phosphorylation cascade thereby attenuating T cell activation (49). The first option suggests that all high-affinity TCR signaling would be attenuated regardless of whether Lck was interacting with CD4; however, IL-2 output reduction in the presence of CD4-Lck sequestration is clearly demonstrated by our intermediate and high affinity CD4+ T cell hybridoma clones. It is also possible that with an increase in affinity and the subsequent decrease in off-rate or increase in half-life, CD4-Lck fails to cycle through the TCR-pMHC synapse, thereby decreasing CD3 phosphorylation and thus downstream activation. Signaling activation is affected by both TCR-pMHCII dwell time and CD4-Lck interactions (70, 88, 89). CD4 increases TCR signaling on low-affinity pMHCII by increasing TCR-CD3 dwell time (39). CD4 dwell time on pMHCII is proportional, yet faster, to TCR dwell time, suggesting that TCR:pMHCII interaction kinetics would directly affect the duration that CD4 molecules cycle through the immunological synapse in a processive-like manner (88). Additionally, compared to coreceptor-bound Lck, CD4-free Lck is phosphorylated more at its Y394 activation site, with higher kinase activity and mobility (81); thus, it may be that if CD4-free Lck is prevented from interacting with the immunological synapse, activation may be reduced. TCR-pMHCII interactions are highly ordered and uniform, increasing the likelihood that the spatial relationship between Lck and the ITAMs of the TCR-SCS or flTCRs are consistent. Thus, kinetic factors, such as TCR-pMHCII affinity would greatly influence the stability of the macrocomplex and consequently the duration of Lck interactions with the ITAMs (39, 90). These kinetics alone could explain the drop in activation observed for our high-affinity, slow off-rate TCR clones. To support this idea, CD8 also acts as a dominant negative inhibitor for ligands that do not recruit fresh CD8 to the TCR-CD3 complex (49).

Previous research suggests that CD4 can send an inhibitory signal independent of Lck *via* post activation antibody-mediated ligation, which attenuates IL-2 production and ongoing activated T cell response (91). This response was also observed in a clonal variant expressing a form of CD4 unable to associate with Lck, suggesting that CD4 has independent inhibitory or regulatory function (91). Furthermore, CD4-mediated inhibition has also been observed during CD4-MHCII interactions leading to a decrease in IL-2 mRNA (91). While we did not seek the source for our Lck-independent CD4 inhibition nor acquire IL-2 mRNA levels, we noted that there was an affinity threshold for this behavior that was independent of MHC interaction, and therefore may be a unique mechanism to that reported in Chervin et al. (49). The affinity threshold for this Lck-independent CD4 inhibition was lower for flTCR (intermediate affinity) than TCR-SCS (high affinity). This may be due to the signaling power of each construct: flTCR-CD3 complexes have 10 ITAMs with 20 tyrosine residues available for phosphorylation, whereas TCR-SCS domains have only 3 ITAMs and 6 tyrosine residues (47, 92, 93). The increased availability of ITAMs per activated Lck may also explain why LLO56_int_ flTCR experienced less IL-2 production inhibition in the presence of CD4—more signal per Lck molecule despite CD4-Lck movement restriction. Whether SCS-TCRs function as dimers is unclear and remains a topic of study (67, 94, 95). It is also curious that the CD4-MHCII interaction supports activation in intermediate affinity TCR-SCS clones, suggesting that while CD4 may not contribute to the overall affinity of TCR-SCS constructs, it may stabilize the interaction between TCR-pMHCII or provide an additional Lck-independent activation signal. The increased interaction stability is more likely as high affinity TCR-SCS IL-2 production is not significantly improved when CD4 interacts with MHCII, suggesting high affinity constructs likely have stable interactions independent of CD4 contributions. Taken together this data suggests an affinity threshold where, up to a point, increased time for CD4-MHCII interactions improves TCR-dependent signaling when it is not Lck-limited, but after a certain affinity point, increased dwell time slows TCR-dependent signaling and positive benefits of CD4-MHCII interactions become redundant.

In addition to the stability challenges presented by scTCR format, the TCR-SCS intracellular format also affected the stability of each TCR. TCR-SCS CD28 format was more stably expressed than other TCR-SCS or flTCR formats, and as noted in other studies, the enhanced surface expression of TCR-SCS CD28 formats *via* increased stability may explain their improved avidity and T cell activation (96–98). However, it is difficult to ascertain whether the increased IL-2 production of TCR-SCS CD28 is due to enhanced stable surface expression or the innate characteristics of CD28-intracellular signaling domains. As observed in numerous antibody-based CAR studies comparing CD28 domains to 4-1BB domains, intracellular signaling domains differentially impact multifactorial T cell response characteristics, including cytokine production (99). For example, CD28-CAR constructs, which can directly bind Lck, are well known for their Lck-binding-dependent enhanced IL-2 production, increased tonic signaling, and subsequent T cell exhaustion compared to 4-1BB CARs (100–103). Thus, the observed increase in IL-2 production for TCR-SCS CD28 constructs may be attributable to the innate characteristics of CD28-intracellular signaling domains rather than increased stable surface expression. As CD28-CARs phosphorylate CD3 more quickly yet do not exceed the levels of CD3 phosphorylation exhibited by 4-1BB CARs, this may be due to signaling intensity (101). Additionally, because CD28 recruits Lck to lipid rafts where it associates with CD4, CD28 may be better able to recruit Lck (104, 105). While TCR-SCS 3^rd^ generation constructs had mixed activation success and overall reduced cytokine production compared to TCR-SCS CD28 constructs, this may be attributable to 3^rd^ generation CAR T cells improved expansion and persistence and may mimic some characteristics of 4-1BB CAR T cells, like reduced cytokine production (106, 107). Our TCR-SCS CD28 constructs demonstrated similarities to antibody-based CD28 CARs, including enhanced tonic signaling in some clones, suggesting that TCR SCS CD28 may also have increased T cell exhaustion. However, unlike CD28 CARs, CD4 expression ameliorated tonic signaling in our TCR-SCS constructs. It will be important to examine the role of CD4 in primary T cells to determine if CD4 prevents exhaustion in clones expressing TCR-SCS CD28 constructs.

CD4^+^ T cells are promising newcomers to immunotherapy. CD4^+^ TCRs convey exquisite target specificity and direct robust immune responses through indirect mechanisms that avoid tumor antigen escape. While much development and thought has been devoted to the activation benefits and off-target effects of increased TCR-pMHC affinity, especially for CD8^+^ TCRs, further TCR-therapeutic development should give consideration to the unique affinity thresholds of TCR-SCS and flTCR formats and the potential inhibitory effects of CD4.

## Data Availability Statement

The original contributions presented in the study are included in the article/**Supplementary Material**. Further inquiries can be directed to the corresponding author.

## Ethics Statement

The animal study was reviewed and approved by Brigham Young University’s Institutional Animal Care and Use Committee (IACUC protocol #18-0708).

## Author Contributions

DJ conceived the experiments. DJ, WM, SM, JF, JH, and TO conducted the experiments. KW and KC provided experimental advice. SP cloned the initial LLO118 and LLO56 constructs in the yeast display constructs and permitted use of previously published data. DJ analyzed data and wrote the manuscript. All authors reviewed the manuscript. All authors contributed to the article and approved the submitted version.

## Funding

This work was supported by a R15 grant to KW (R0102063) from the National Institutes of Health, Simmons Center for Cancer Research Summer Fellowships and Graduate Student Fellowship grants at Brigham Young University to DJ.

## Conflict of Interest

The authors declare that the research was conducted in the absence of any commercial or financial relationships that could be construed as a potential conflict of interest.

## Supplementary Material

Supplementary Figure 1 LLO118 and LLO56 single-chain TCRs stabilizing mutations. Wild type templates (LLO118_WT_ and LLO56_WT_) compared to stabilized single-chain TCR (scTCR) templates (LLO118_low_ and LLO56_low_). The original LLO56_WT_ scTCR template included mutations of the amino acid K42βG, H36αY and S74αT (highlighted gray) known to enhance surface display levels in related TCRs (108). Stability mutations selected by random mutagenesis and directed evolution are marked in red. Boxed amino acids show joint LLO118_low_ and LLO56_low_ selection (K42βG and T93βA), and mutations in another known stability hotspot (L45αI and I49αM) are unmarked. LLO118_low_ independently selected I115αK, and LLO56_low_ selected T93βA and S9αT.

Supplementary Figure 2 Protein sequence map for 4-1BB SCS-TCR formats for **(A)** LLO56_low_ and LLO56_int_, and **(B)** LLO118_low_, LLO118_int_, and LLO118_high_. Protein fragments are delineated by color bar above the amino acid sequence. Stability mutations (highlighted grey or red font) are marked as indicated in **Supplemental Figure 1**. CDR3β mutations for additional clones are listed below the sequence map.

Supplementary Figure 3 Protein sequence map for CD28 SCS-TCR formats for **(A)** LLO56_low_ and LLO56_int_, and **(B)** LLO118_low_, LLO118_int_, and LLO118_high_. Protein fragments are delineated by color bar above the amino acid sequence. Stability mutations (highlighted grey or red font) are marked as indicated in **Supplemental Figure 1**. CDR3β mutations for additional clones are listed below the sequence map.

Supplementary Figure 4 Protein sequence map for 3^rd^ gen SCS-TCR formats for **(A)** LLO56_low_ and LLO56_int_, and **(B)** LLO118_low_, LLO118_int_, and LLO118_high_. Protein fragments are delineated by color bar above the amino acid sequence. Stability mutations (highlighted grey or red font) are marked as indicated in **Supplemental Figure 1**. CDR3β mutations for additional clones are listed below the sequence map.

Supplementary Figure 5 Protein sequence map for flTCR formats for LLO56_low_ and LLO56_int_. Protein fragments are delineated by color bar above the amino acid sequence. Stability mutations were removed for flTCR constructs. CDR3β mutations for additional clones are listed below the sequence map.

Supplementary Figure 6 GFP expression levels for CD4^-^ and CD4^+^ clones. GFP MFI is significantly different for most scTCR and flTCR constructs between CD4^-^ and CD4^+^ pairs including LLO56_low_ 4-1BB (p < 0.0001), LLO56_int_ 4-1BB (p < 0.0001), LLO56_low_ CD28 (p = 0.0016), LLO56_int_ CD28 (p = 0.0001), LLO56_low_ 3^rd^ gen (p = 0.0054), LLO56_int_ 3^rd^ gen (p < 0.0001), LLO56_low_ flTCR (p = 0.0058), LLO118_low_ 4-1BB (p < 0.0001), LLO118_int_ 4-1BB (p < 0.0001), LLO118_low_ CD28 (p < 0.0001), LLO118_int_ CD28 (p = 0.0012), LLO118_int_ 3^rd^ gen (p < 0.0001), and LLO118_high_ 3^rd^ gen (p < 0.0001).

Supplementary Figure 7 Comparison of IL-2 production at 10^-3^ M peptide stimulation. Analysis of levels of IL-2 production from **Figure 5** at 10^-3^ M peptide stimulation. IL-2 production between 58-/-CD4- affinity clones was significantly different for LLO56low and LLO56int 4-1BB (p = 0.0211), LLO56low and LLO56int CD28 (p = 0.0039) and LLO56low and LLO56int flTCR (p = 0.0033), LLO118low and LLO118int 3rd gen (p = 0.0006), LLO118low and LLO118high 3rd gen (p = 0.0070), LLO118low and LLO118int 4-1BB (p = 0.0441), LLO118low and LLO118high 4-1BB (p = 0.0115), LLO118low and LLO118int CD28 (p = 0.0489), LLO118low and LLO118high CD28 (p < 0.0001), and LLO118int and LLO118high CD28 (p < 0.0001).

Supplementary Figure 8 CD4 is not expressed in CD4^-^ SCS-TCR, flTCR and base 58^-/-^ hybridoma cell lines. Twenty-five thousand cells were stained with antibody mix for 10 mins. 10,000 events collected *via* flow cytometry. **(A)** Isotype stain LLO56_high_ CD28, LLO56_high_ flTCR, and base 58^-/-^ T cell hybridoma lines not expressing TCRs were stained with antibody mix [anti-Vβ2 PE, anti-Vα APC, and anti-F4/80 PE Cy7 (rat IgG2, Fisher Scientific)]. Histograms display anti-F4/80 PE-Cy7: Cells only (grey), CD4^-^ clones (green), and all CD4^+^ clones (red) including CD4^+^, CD4T^+^ and CD4T^+^ Δbind. **(B)** CD4 stain of LLO56_high_ CD28, LLO56_high_ flTCR, and base 58^-/-^ T cell hybridoma lines not expressing TCRs were stained with antibody mix [anti-Vβ2 PE, anti-Vα APC, and anti-CD4 PE Cy7 (rat IgG2, Fisher Scientific)]. Histograms display anti-F4/80 PE-Cy7: Cells only (grey), CD4^-^ clones (blue), and all CD4^+^ clones (red) including CD4^+^, CD4T^+^ and CD4T^+^ Δbind. **(C)** Comparison of isotype stain (green) and CD4 stain (blue) for CD4^-^.

Supplementary Figure 9 GFP expression levels for SCS constructs **(A)** GFP expression by construct in order of 58^-/-^ CD4^-^, 58^-/-^ CD4^+^, 58^-/-^ CD4T^+^, 58^-/-^ CD4T^+^ Δbind T cell hybridomas. Both CD4T^+^ and CD4T^+^ Δbind and TCRs were inserted into the cell lines *via* the vector pMIGII which uses GFP as a reporter gene. Thus, the GFP expression for all CD4T^+^ and CD4T^+^ Δbind cell lines are significantly higher than CD4^-^ and CD4^+^ cell lines. LLO56_low_ (pink colors), LLO56_int_ (red colors), LLO118_int_ (light blue colors), LLO118_high_ (dark blue colors). Hatched lines indicate CD4-MHC interactions. LLO56_low_ 4-1BB (p < 0.0001), LLO56_int_ 4-1BB (p < 0.0001), LLO56_low_ CD28 (p < 0.0001), LLO56_int_ CD28 (p < 0.0001), LLO118_int_ 3^rd^ gen (p < 0.0001), LLO118_high_ 3^rd^ gen (p < 0.0001), LLO118_int_ CD28 (p < 0.0001), and LLO118_high_ CD28 (p < 0.0001). **(B)** Stable expression of Vβ2 by construct. LLO56_low_ CD28 (p < 0.0001) and LLO56_int_ CD28 (p < 0.0001) **(C)** To ascertain whether avidity was maintained within each TCR-construct despite CD4 variations, each cell line was tested at sub-saturating tetramer concentrations: LLO56_low_ (10^-8^ M), LLO56_int_ (5 x 10^-10^ M), LL0118_int_ (10^-9^ M), and LL0118_high_ (5 x 10^-10^ M). Colors and order as found in **(B)**. LLO118_int_ CD28 (p = 0.033). Groups analyzed for differences with one-way ANOVA.

Supplementary Table 1 Summary of T cell responses of LLO56 and LLO118 to antigen adapted from Persaud *et al*. LLO118 and LLO56 differentially respond to activation by peptide LLO_190-205_. LLO118 and LLO56 have similar proliferation responses to LLO_190-205_ *in vitro*, but *in vivo* LLO118 has a more robust primary response while LLO56 has a more robust secondary response. LLO56 produces more IL-2 *in vitro* and undergoes higher rates of apoptosis during the primary response than LLO118. Surface plasma resonance measurements of LLO56 and LLO118 dissociation constants are 27.4 and 28.3 µM, respectively (41, 42).
