# Supplementary material for: CD4 Inhibits Helper T Cell Activation at Lower Affinity Threshold for Full-Length T Cell Receptors Than Single Chain Signaling Constructs
Source: Front Immunol. 2021 Jan 19;11:561889. doi: 10.3389/fimmu.2020.561889 (PMC7851051; doi:10.3389/fimmu.2020.561889)

Supplemental Table S1. Response of LLO56 and LLO118 T cells to antigen

Parameter	LLO56	LLO118
Primary response *in vivo*	+	+++
Secondary response *in vivo*	+++	+
Proliferation in response to peptide $LLO_{190\text{-}205}$ *in vitro*	+	+
Proliferation in response to *Listeria monocytogenes in vitro*	+	+
Rate of apoptosis *in vivo*	+++	+
IL-2 production *in vitro*	++	+
K_D (uM) $LLO_{190\text{-}205}$ (surface plasmon resonance)	+	+

Supplemental Figure S1

Vβ2

Position	Region	LLO56$_{WT}$	LLO56$_{low}$	LLO118$_{WT}$	LLO118$_{low}$
1		V	-	V	-
2		T	-	T	-
3		L	-	L	-
4		L	-	L	-
5		E	-	E	-
6		Q	-	Q	-
7		N	-	N	-
8		P	-	P	-
9		R	-	R	-
10		W	-	W	-
11		R	-	R	-
12		L	-	L	-
13		V	-	V	-
14		P	-	P	-
15		R	-	R	-
16		G	-	G	-
17		Q	-	Q	-
18		A	-	A	-
19		V	-	V	-
20		N	-	N	-
21		L	-	L	-
22		R	-	R	-
23		C	-	C	-
24		I	-	I	-
25		L	-	L	-
26	CDR 1	K	-	K	-
27	CDR 1	N	-	N	-
28	CDR 1	S	-	S	-
29	CDR 1	Q	-	Q	-
30	CDR 1	Y	-	Y	-
31	CDR 1	P	-	P	-
32	CDR 1	W	-	W	-
33	CDR 1	M	-	M	-
34		S	-	S	-
35		W	-	W	-
36		Y	-	Y	-
37		Q	-	Q	-
38		Q	-	Q	-
39		D	-	D	-
40		L	-	L	-
41		Q	-	Q	-
42		G	-	K	G
43		Q	-	Q	-
44		L	-	L	-
45		Q	-	Q	-
46		W	-	W	-
47		L	-	L	-
48	CDR 2	F	-	F	-
49	CDR 2	T	-	T	-
50	CDR 2	L	-	L	-
51	CDR 2	R	-	R	-
52	CDR 2	N	-	S	-
53	CDR 2	P	-	P	-
54	CDR 2	G	-	G	-
55	CDR 2	D	-	D	-
56	CDR 2	K	-	K	-
57		E	-	E	-
58		V	-	V	-
59		K	-	K	-

Vβ2 cont.

Position	Region	LLO56$_{WT}$	LLO56$_{low}$	LLO118$_{WT}$	LLO118$_{low}$
60		S	-	S	-
61		L	-	L	-
62		P	-	P	-
63		G	-	G	-
64		A	-	A	-
65		_	_	_	_
66		D	-	D	-
67		Y	-	Y	-
68		L	-	L	-
69		A	-	A	-
70	HV4	T	-	T	-
71	HV4	R	-	R	-
72	HV4	V	-	V	-
73	HV4	T	-	T	-
74	HV4	D	-	D	-
75	HV4	T	-	T	-
76	HV4	E	-	E	-
77	HV4	L	-	L	-
78		R	-	R	-
79		L	-	L	-
80		Q	-	Q	-
81		V	-	V	-
82		A	-	A	-
83		N	-	N	-
84		M	-	M	-
85		S	-	S	-
86		Q	-	Q	-
87		G	-	G	-
88		R	-	R	-
89		T	-	T	-
90		L	-	L	-
91		Y	-	Y	-
92		C	-	C	-
93		T	A	T	A
94		C	-	C	-
95		S	-	S	-
96	CDR 3	A	-	G	-
97	CDR 3	D	-	G	-
98	CDR 3	V	-	G	-
99	CDR 3	G	-	I	-
100	CDR 3	_	-	_	_
101	CDR 3	_	-	_	_
102	CDR 3	_	-	_	_
103	CDR 3	_	-	S	-
104	CDR 3	_	-	Q	-
105	CDR 3	_	-	N	-
106	CDR 3	_	-	T	-
107	CDR 3	V	-	L	-
108	CDR 3	F	-	Y	-
109		F	-	F	-
110		G	-	G	-
111		K	-	A	-
112		G	-	G	-
113		T	-	T	-
114		R	-	R	-
115		L	-	L	-
116		T	-	S	-
117		V	-	V	-
118		V	-	L	-

Vα2

Position	Region	LLO56$_{WT}$	LLO56$_{low}$	LLO118$_{WT}$	LLO118$_{low}$
1		Q	-	Q	-
2		Q	-	Q	-
3		V	-	V	-
4		R	-	R	-
5		Q	-	Q	-
6		S	-	S	-
7		P	-	P	-
8		Q	-	Q	-
9		S	T	S	-
10		L	-	L	-
11		T	-	T	-
12		V	-	V	-
13		W	-	W	-
14		E	-	E	-
15		G	-	G	-
16		E	-	E	-
17		T	-	T	-
18		A	-	A	-
19		I	-	I	-
20		L	-	L	-
21		N	-	N	-
22		C	-	C	-
23		S	-	S	-
24	CDR 1	Y	-	Y	-
25	CDR 1	E	-	E	-
26	CDR 1	D	-	D	-
27	CDR 1	S	-	S	-
28	CDR 1	T	-	T	-
29	CDR 1	F	-	F	-
30	CDR 1	D	-	D	-
31	CDR 1	Y	-	Y	-
32		F	-	F	-
33		P	-	S	-
34		W	-	W	-
35		Y	-	Y	-
36		Y	-	H	-
37		Q	-	Q	-
38		F	-	F	-
39		P	-	P	-
40		G	-	G	-
41		E	-	E	-
42		S	-	S	-
43		P	-	P	-
44		A	-	A	-
45		L	-	L	I
46		L	-	L	-
47		I	-	I	-
48	CDR 2	A	-	A	-
49	CDR 2	I	M	I	-
50	CDR 2	R	-	R	-
51	CDR 2	P	-	P	-
52	CDR 2	V	-	V	-
53	CDR 2	S	-	S	-
54	CDR 2	N	-	N	-
55	CDR 2	K	-	K	-
56		K	-	K	-
57		E	-	E	-
58		D	-	D	-

Vα2 cont.

Position	Region	LLO56$_{WT}$	LLO56$_{low}$	LLO118$_{WT}$	LLO118$_{low}$
59		G	-	G	-
60		R	-	R	-
61		F	-	F	-
62		T	-	T	-
63		I	-	I	-
64		F	-	F	-
65		F	-	F	-
66		N	-	N	-
67		K	-	K	-
68		R	-	R	-
69		E	-	E	-
70		_	-	_	-
71		K	-	K	-
72		K	-	K	-
73		F	-	F	-
74		T	-	S	-
75		L	-	L	-
76		H	-	H	-
77		I	-	I	-
78		A	-	A	-
79		D	-	D	-
80		S	-	S	-
81		Q	-	Q	-
82		P	-	P	-
83		G	-	G	-
84		D	-	D	-
85		S	-	S	-
86		A	-	A	-
87		T	-	T	-
88		Y	-	Y	-
89		F	-	F	-
90		C	-	C	-
91		A	-	A	-
92		A	-	A	-
93	CDR 3	K	-	S	-
94	CDR 3	_	-	_	-
95	CDR 3	_	-	_	-
96	CDR 3	_	-	_	-
97	CDR 3	_	-	_	-
98	CDR 3	D	-	D	-
99	CDR 3	T	-	T	-
100	CDR 3	G	-	G	-
101	CDR 3	N	-	N	-
102	CDR 3	Y	-	Y	-
103	CDR 3	K	-	K	-
104	CDR 3	Y	-	Y	-
105		V	-	V	-
106		F	-	F	-
107		G	-	G	-
108		A	-	A	-
109		G	-	G	-
110		T	-	T	-
111		R	-	R	-
112		L	-	L	-
113		K	-	K	-
114		V	-	V	-
115		I	-	I	K
116		A	-	A	-

Supplemental Figure S2

A

LLO56$_{low}$ 4-1BB

M33 3rd gen signal peptide | Vβ2

M L L A L L P V L G I H F V L R D A Q A H M V T L L E Q N P R W R L V P R G Q A V N L R C I L K N S

Q Y P W M S W Y Q Q D L Q **G** Q L Q W L F T L R N P G D K E V K S L P G A D Y L A T R V T D T E L R L

CDR3 | Linker

Q V A N M S Q G R T L Y C **A** C S A D V G V F F G K G T R L T V V G S A D D A K K D A A K K D D A S Q

Vα2

Q Q V R Q S P Q **T** L T V W E G E T A I L N C S Y E D S T F D Y F P W Y **Y** Q F P G E S P A L L I A **M** R

P V S N K K E D G R F T I F F N K R E K K F **T** L H I A D S Q P G D S A T Y F C A A K D T G N Y K Y V

CD8 hinge

F G A G T R L K V I A K V N S T T T K P V L R T P S P V H P T G T S Q P Q R P E D C R P R G S V K G

4-1BB

T G L D F A C D F W A L V V V A G V L F C Y G L L V T V A L C V I W T S K W I R K K F P H I F K Q P

CD3ζ

F K K T T G A A Q E E D A C S C R C P Q E E E G G G G Y E L R A K F S R S A E T A A N L Q D P N Q

L Y N E L N L G R R E E Y D V L E K K R A R D P E M G G K Q Q R R R N P Q E G V Y N A L Q K D K M A

E A Y S E I G T K G E R R R G K G H D G L Y Q G L S T A T K D T Y D A L H M Q T L A P R * *

CDR3β

LLO56$_{int}$ A **P** **W** **D** V **G**

B

LLO118$_{int}$ 4-1BB

M33 3rd gen signal peptide | Vβ2

M L L A L L P V L G I H F V L R D A Q A H *H* *M* *V* T L L E Q N P R W R L V P R G Q A V N L R C I L K N

S Q Y P W M S W Y Q Q D L Q **G** Q L Q W L F T L R S P G D K E V K S L P G A D Y L A T R V T D T E L R

CDR3β | Linker

L Q V A N M S Q G R T L Y C **A** C S G **W** G **S** **N** Q N T L Y F G A G T R L S V L G S A D D A K K D A A K K

Vα2

D D A S *Q* *Q* *Q* V R Q S P Q S L T V W E G E T A I L N C S Y E D S T F D Y F S W Y H Q F P G E S P A L

L I A I R P V S N K K E D G R F T I F F N K R E K K F S L H I A D S Q P G D S A T Y F C A A S D T G

CD8 hinge

N Y K Y V F G A G T R L K V **K** A *R* *S* *D* *N* *N* *S* *V* *D* *V* *T* *K* *S* *T* *L* *F* *P* *L* *Y* *F* K V N S T T T K P V L R T P S

4-1BB

P V H P T G T S Q P Q R P E D C R P R G S V K G T G L D F A C D F W A L V V V A G V L F C Y G L L V

T V A L C V I W T S K W I R K K F P H I F K Q P F K K T T G A A Q E E D A C S C R C P Q E E E G G G

CD3ζ

G G Y E L R A K F S R S A E T A A N L Q D P N Q L Y N E L N L G R R E E Y D V L E K K R A R D P E M

G G K Q Q R R R N P Q E G V Y N A L Q K D K M A E A Y S E I G T K G E R R R G K G H D G L Y Q G L S

T A T K D T Y D A L H M Q T L A P R * *

CDR3β

LLO118$_{low}$ G G G I S Q N T L Y

LLO118$_{high}$ G **W** G **S** **N** **P** **Q** **Q** **W** **Q**

Supplemental Figure S3

A **LLO56$_{low}$ CD28**

M33 3rd gen signal peptide | Vβ2

M L L A L L P V L G I H F V L R D A Q A H M V T L L E Q N P R W R L V P R G Q A V N L R C I L K N S

Q Y P W M S W Y Q Q D L Q G Q L Q W L F T L R N P G D K E V K S L P G A D Y L A T R V T D T E L R L

CDR3 | Linker

Q V A N M S Q G R T L Y C A C S A D V G V F F G K G T R L T V V G S A D D A K K D A A K K D D A S Q

Vα2

Q Q V R Q S P Q T L T V W E G E T A I L N C S Y E D S T F D Y F P W Y Y Q F P G E S P A L L I A M R

P V S N K K E D G R F T I F F N K R E K K F T L H I A D S Q P G D S A T Y F C A A K D T G N Y K Y V

CD8 hinge

F G A G T R L K V I A K V N S T T T K P V L R T P S P V H P T G T S Q P Q R P E D C R P R G S V K G

CD28

T G L D F A C D S S P K L F W A L V V V A G V L F C Y G L L V T V A L C V I W T N S R R N R L L Q V

CD3ζ

T T M N M T P R R P G L T R K P Y Q P Y A P A R D F A A Y R P A H A R A K F S R S A E T A A N L Q D

P N Q L Y N E L N L G R R E E Y D V L E K K R A R D P E M G G K Q Q R R R N P Q E G V Y N A L Q K D

K M A E A Y S E I G T K G E R R R G K G H D G L Y Q G L S T A T K D T Y D A L H M Q T L A P R * *

CDR3β

LLO56$_{int}$ A **P** **W D** V **G**

B **LLO118$_{int}$ CD28**

M33 3rd gen signal peptide | Vβ2

M L L A L L P V L G I H F V L R D A Q A H *H M V* T L L E Q N P R W R L V P R G Q A V N L R C I L K N

S Q Y P W M S W Y Q Q D L Q G Q L Q W L F T L R S P G D K E V K S L P G A D Y L A T R V T D T E L R

CDR3β | Linker

L Q V A N M S Q G R T L Y C A C S G **W** G **S** **N** Q N T L Y F G A G T R L S V L G S A D D A K K D A A K K

Vα2

D D A S *Q Q Q* V R Q S P Q S L T V W E G E T A I L N C S Y E D S T F D Y F S W Y H Q F P G E S P A L

L I A I R P V S N K K E D G R F T I F F N K R E K K F S L H I A D S Q P G D S A T Y F C A A S D T G

CD8 hinge

N Y K Y V F G A G T R L K V K *A R S D N N S V D V T K S T L F P L Y F* K V N S T T T K P V L R T P S

CD28

P V H P T G T S Q P Q R P E D C R P R G S V K G T G L D F A C D S S P K L F W A L V V V A G V L F C

Y G L L V T V A L C V I W T N S R R N R L L Q V T T M N M T P R R P G L T R K P Y Q P Y A P A R D F

CD3ζ

A A Y R P A H A R A K F S R S A E T A A N L Q D P N Q L Y N E L N L G R R E E Y D V L E K K R A R D

P E M G G K Q Q R R R N P Q E G V Y N A L Q K D K M A E A Y S E I G T K G E R R R G K G H D G L Y Q

G L S T A T K D T Y D A L H M Q T L A P R * *

CDR3β

LLO118$_{low}$ G G G I S Q N T L Y

LLO118$_{high}$ G **W** G **S N P Q Q W Q**

Supplemental Figure S4

A

LLO56$_{low}$ 3rd gen

M33 3rd gen signal peptide | Vβ2

M L L A L L P V L G I H F V L R D A Q A H M V T L L E Q N P R W R L V P R G Q A V N L R C I L K N S

Q Y P W M S W Y Q Q D L Q G Q L Q W L F T L R N P G D K E V K S L P G A D Y L A T R V T D T E L R L

CDR3 | Linker

Q V A N M S Q G R T L Y C A C S A D V G V F F G K G T R L T V V G S A D D A K K D A A K K D D A S Q

Vα2

Q Q V R Q S P Q T L T V W E G E T A I L N C S Y E D S T F D Y F P W Y Y Q F P G E S P A L L I A M R

P V S N K K E D G R F T I F F N K R E K K F T L H I A D S Q P G D S A T Y F C A A K D T G N Y K Y V

CD8 hinge

F G A G T R L K V I A K V N S T T T K P V L R T P S P V H P T G T S Q P Q R P E D C R P R G S V K G

CD28

T G L D F A C D S S P K L F W A L V V V A G V L F C Y G L L V T V A L C V I W T N S R R N R L L Q V

4-1BB

T T M N M T P R R P G L T R K P Y Q P Y A P A R D F A A Y R P A H A F W A L V V V A G V L F C Y G L

L V T V A L C V I W T S K W I R K K F P H I F K Q P F K K T T G A A Q E E D A C S C R C P Q E E E G

CD3ζ

G G G G Y E L R A K F S R S A E T A A N L Q D P N Q L Y N E L N L G R R E E Y D V L E K K R A R D P

E M G G K Q Q R R R N P Q E G V Y N A L Q K D K M A E A Y S E I G T K G E R R R G K G H D G L Y Q G

L S T A T K D T Y D A L H M Q T L A P R * *

CDR3β

LLO56$_{int}$ A **P** **W** **D** V **G**

B

LLO118$_{int}$ 3rd gen

M33 3rd gen signal peptide | Vβ2

M L L A L L P V L G I H F V L R D A Q A H H M V T L L E Q N P R W R L V P R G Q A V N L R C I L K N

S Q Y P W M S W Y Q Q D L Q G Q L Q W L F T L R S P G D K E V K S L P G A D Y L A T R V T D T E L R

CDR3β | Linker

L Q V A N M S Q G R T L Y C A C S G **W** G **S** **N** Q N T L Y F G A G T R L S V L G S A D D A K K D A A K K

Vα2

D D A S *Q Q Q* V R Q S P Q S L T V W E G E T A I L N C S Y E D S T F D Y F S W Y H Q F P G E S P A L

L I A I R P V S N K K E D G R F T I F F N K R E K K F S L H I A D S Q P G D S A T Y F C A A S D T G

CD8 hinge

N Y K Y V F G A G T R L K V K A *R S D N N S V D V T K S T L F P L Y F* K V N S T T T K P V L R T P S

CD28

P V H P T G T S Q P Q R P E D C R P R G S V K G T G L D F A C D S S P K L F W A L V V V A G V L F C

Y G L L V T V A L C V I W T N S R R N R L L Q V T T M N M T P R R P G L T R K P Y Q P Y A P A R D F

4-1BB

A A Y R P A H A F W A L V V V A G V L F C Y G L L V T V A L C V I W T S K W I R K K F P H I F K Q P

CD3ζ

F K K T T G A A Q E E D A C S C R C P Q E E E G G G G G Y E L R A K F S R S A E T A A N L Q D P N Q

L Y N E L N L G R R E E Y D V L E K K R A R D P E M G G K Q Q R R R N P Q E G V Y N A L Q K D K M A

E A Y S E I G T K G E R R R G K G H D G L Y Q G L S T A T K D T Y D A L H M Q T L A P R * *

CDR3β

LLO118$_{low}$ G G G I S Q N T L Y

LLO118$_{high}$ G **W** G **S** **N** **P** **Q** **Q** **W** **Q**

Supplemental Figure S5

$LLO56_{low}$ flTCR

Vα2 Signal peptide Vα2

M D K I L T A S F L L L G L H L A G V S G Q Q Q Q V R Q S P Q S L T V W E G E T A I L N C S Y E D S

T F D Y F P W Y Y Q F P G E S P A L L I A I R P V S N K K E D G R F T I F F N K R E K K F T L H I A

Cα2

D S Q P G D S A T Y F C A A K D T G N Y K Y V F G A G T R L K V I A I Q N P E P A V Y Q L K D P R S

Q D S T L C L F T D F D S Q I N V P K T M E S G T F I T D K C V L D M K A M D S K S N G A I A W S N

Q T S F T C Q D I F K E T N A T Y P S S D V P C D A T L T E K S F E T D M N L N F Q N L S V M G L R

P2a Vβ2 leader sequence

I L L L K V A G F N L L M T L R L W S S G S G A T N F S L L K Q A G D V E E N P G P M W Q F C I L C

Vβ2

L C V L M A S V A T D H M V T L L E Q N P R W R L V P R G Q A V N L R C I L K N S Q Y P W M S W Y Q

Q D L Q G Q L Q W L F T L R N P G D K E V K S L P G A D Y L A T R V T D T E L R L Q V A N M S Q G R

CDR3β Cβ

T L Y C T C S A D V G V F F G K G T R L T V V D L R N V T P P K V S L F E P S K A E I A N K Q K A T

L V C L A R G F F P D H V E L S W W V N G K E V H S G V C T D P Q A Y K E S N Y S Y C L S S R L R V

S A T F W H N P R N H F R C Q V Q F H G L S E E D K W P E G S P K P V T Q N I S A E A W G R A D C G

I T S A S Y H Q G V L S A T I L Y E I L L G K A T L Y A V L V S G L V L M A M V K K K N S G S G * *

CDR3β

$LLO56_{int}$ A **P** **W** **D** V **G**

Supplemental Figure S6

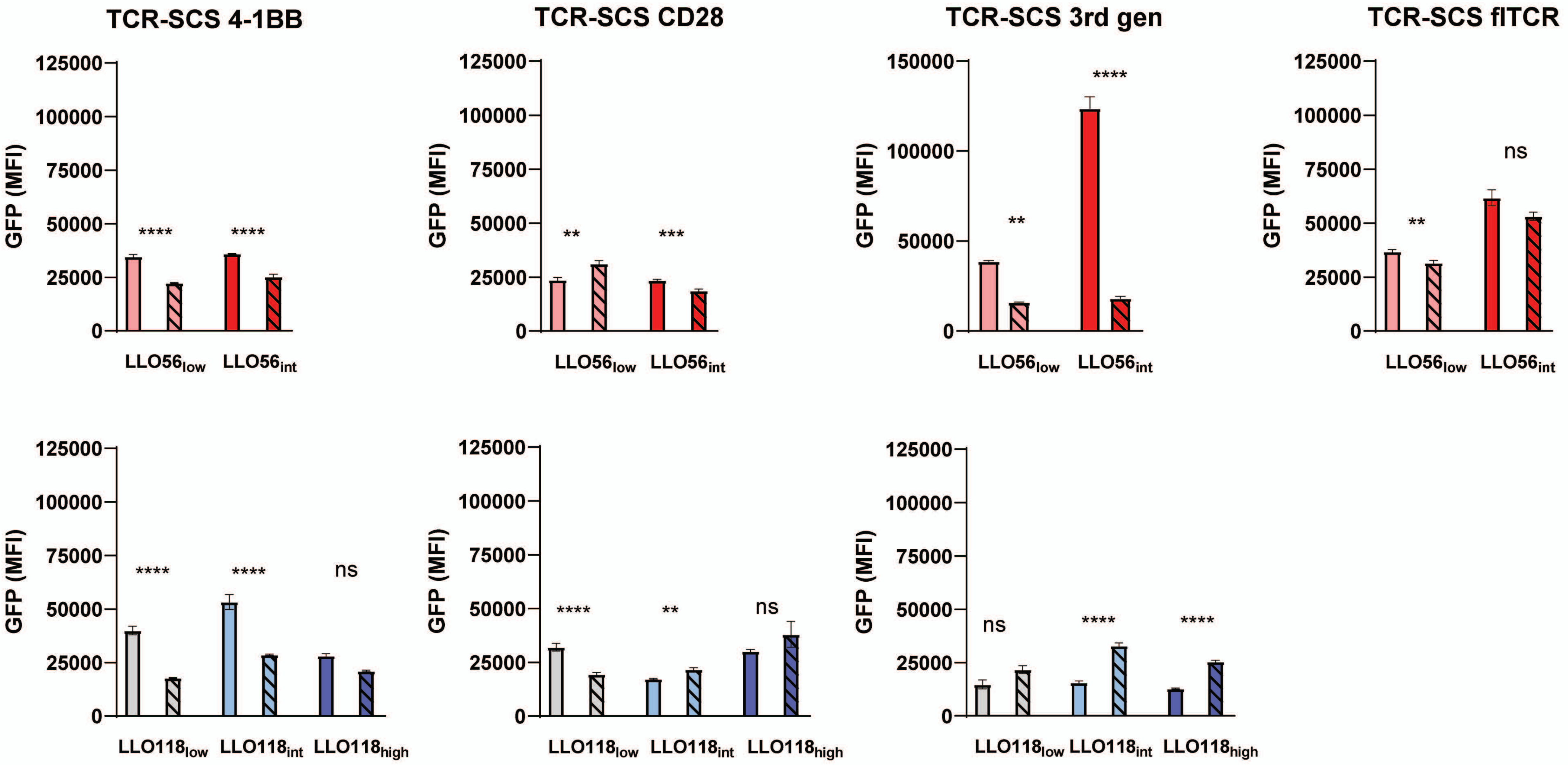

Supplemental Fig. S7

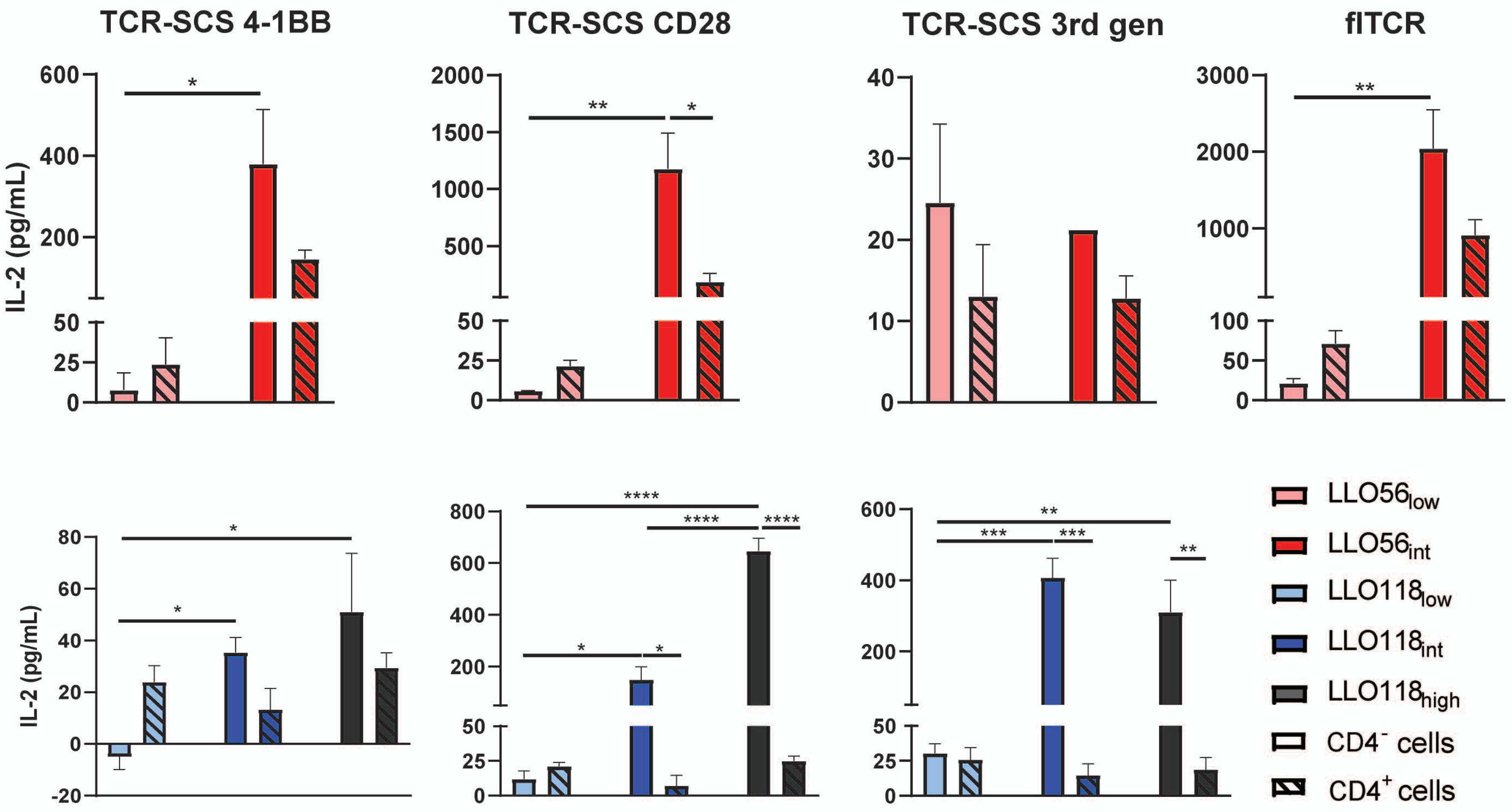

Supplemental Figure 8

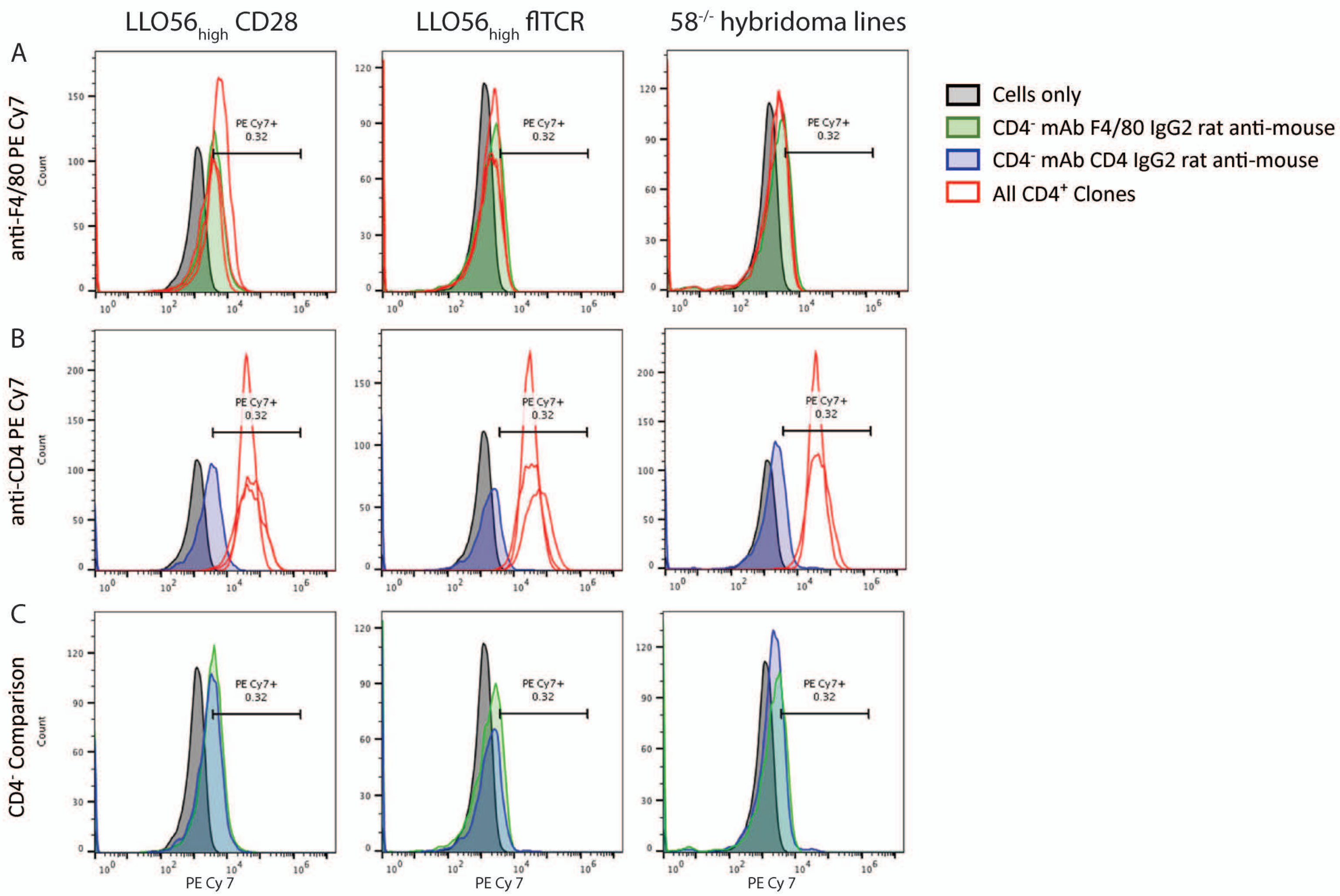

Supplemental Fig. S9

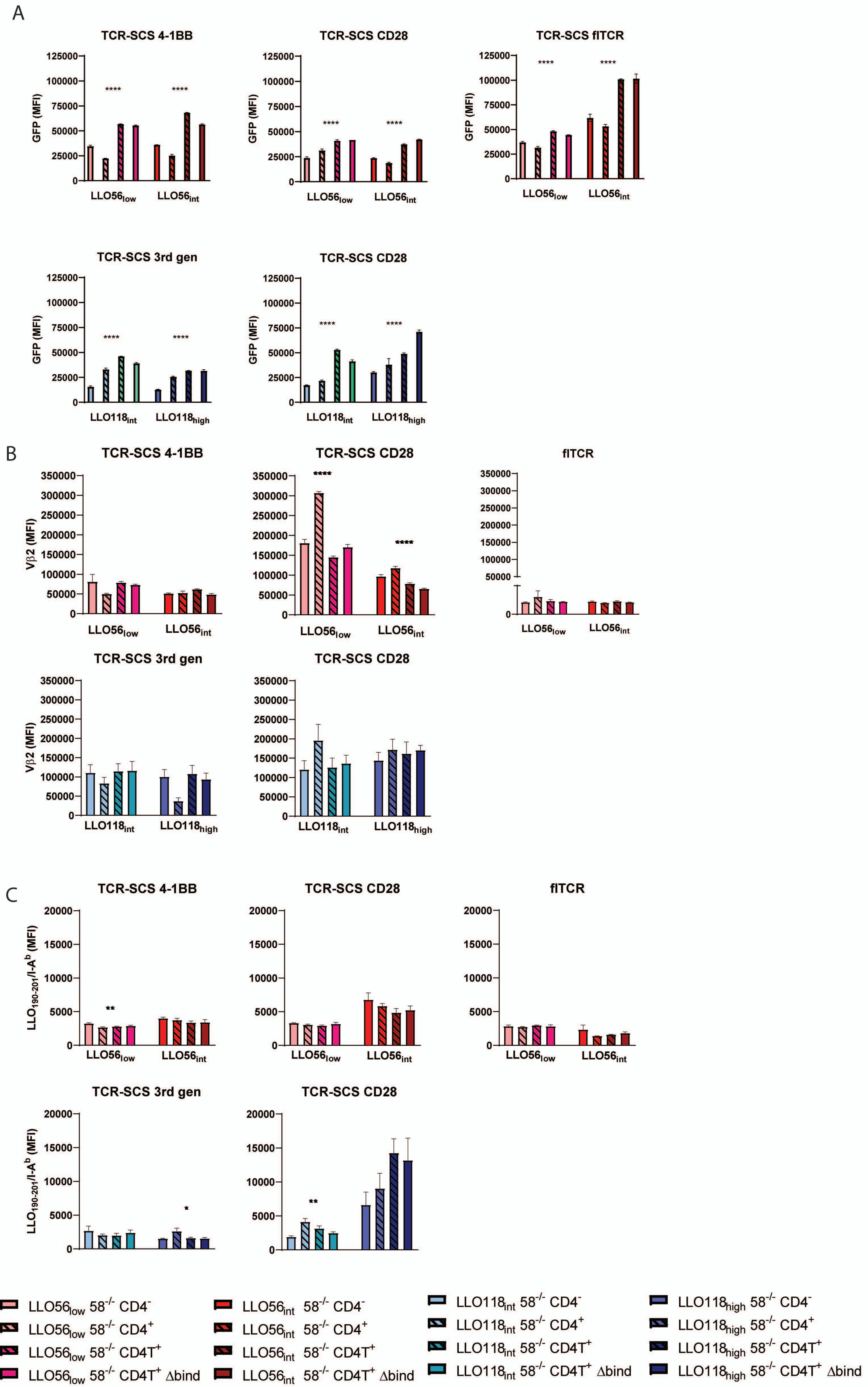

Supplement: Supplementary Figure 1 — LLO118 and LLO56 single-chain TCRs stabilizing mutations. Wild type templates (LLO118WT and LLO56WT) compared to stabilized single-chain TCR (scTCR) templates (LLO118low and LLO56low). The original LLO56WT scTCR template included mutations of the amino acid K42βG, H36αY and S74αT (highlighted gray) known to enhance surface display levels in related TCRs (108). Stability mutations selected by random mutagenesis and directed evolution are marked in red. Boxed amino acids show joint LLO118low and LLO56low selection (K42βG and T93βA), and mutations in another known stability hotspot (L45αI and I49αM) are unmarked. LLO118low independently selected I115αK, and LLO56low selected T93βA and S9αT. [file DataSheet_1.pdf]
